# From Waste to Value-Added Resource: A Strategic Review of Recycling and Regeneration Pathways for Fiber-Reinforced Polymer Waste

**DOI:** 10.3390/polym18172038

**Published:** 2026-08-22

**Authors:** Yi Liu, Yingfang Fan, Lei Wang, Wenjie Qi

**Affiliations:** 1 College of Transportation Engineering, Dalian Maritime University, Dalian 116026, China; liuyimew@163.com (Y.L.); 0120240121@dlmu.edu.cn (L.W.); qwj9089@163.com (W.Q.); 2 Liaoning Key Laboratory of Coastal Bridge and Tunnel Engineering, Dalian Maritime University, Dalian 116026, China

**Keywords:** fiber reinforced polymer, cementitious composite, recycling, regenerated composite

## Abstract

The rapid expansion of fiber-reinforced polymers (FRPs) in wind energy, transportation, and aerospace is generating increasing amounts of accompanied waste, making effective valorization essential to a circular economy. This review compares FRP recovery technologies in terms of recovered-fiber quality, operating conditions, post-treatment, environmental impacts, and industrial applicability. Then, it also examines direct reuse, FRP remanufacturing, and reuse in cementitious composites. Quantitative synthesis indicates that high-quality recycled carbon fibers (rCFs) generally retain more than 90% of their original strength, whereas mechanically recovered glass fibers (rGFs) typically retain approximately 70–90%. The preferred pathway depends on the intrinsic value, damage state, morphology, and residual properties. Components with sufficient residual capacity should be directly reused; high-quality fibers are better suited to polymer remanufacturing; and heterogeneous or lower-grade glass-FRP (GFRP) fractions are more compatible with cementitious applications, where mechanically recycled GFRP can provide interfacial bond strengths comparable to conventional engineering macrofibers. Future research should establish quantitative links among recovered material quality, processing, interfacial behavior, and end-use performance, while adopting consistent environmental and economic assessment boundaries. A graded utilization framework is therefore required to support both large-scale and value-added reuse of FRP waste.

## 1. Introduction

Fiber-reinforced polymers (FRP) are high-performance materials composed of reinforced fibers, such as glass, carbon, aramid fibers, and various synthetic and natural fibers, embedded in a polymer matrix, and are characterized by high strength, light weight, electromagnetic transparency, electrical insulation, and corrosion resistance [1]. Since the 1940s, glass-FRP (GFRP), carbon-FRP (CFRP), aramid-FRP, and basalt-FRP have been widely used in construction, automotive, energy, and other sectors [2,3,4,5,6,7,8,9,10,11,12]. With the rapid expansion of the global FRP market, which is projected to reach USD 610.99 billion by 2035 [13], the volume of FRP waste generated at the end-of-life (EoL) stage has increased sharply, while its polymer matrix remains difficult to degrade naturally [14]. Meanwhile, the waste mother liquor and sludge generated from intermediate products required for FRP production, such as polymer matrices and plasticizers, are also classified as hazardous wastes [15]. Therefore, the recycling and reutilization of FRP has become an important issue of resource, environmental, and industrial significance.

**Figure 1:**
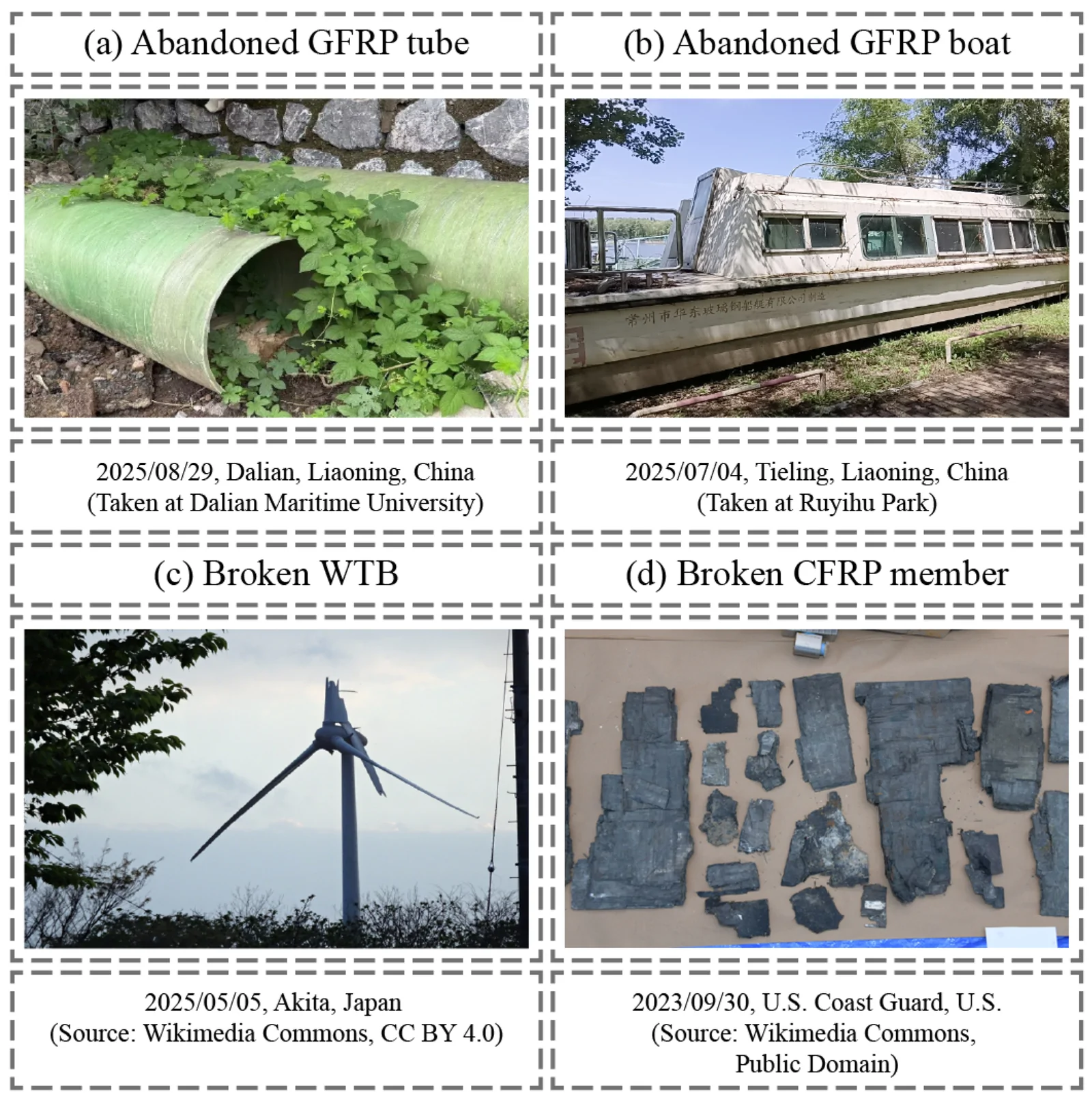
Examples of abandoned and damaged FRP components: (**a**) abandoned GFRP tube; (**b**) abandoned GFRP boat; (**c**) broken wind turbine blade (WTB) [24]; (**d**) broken CFRP member [25].

As depicted in Figure 1, currently, FRP waste mainly originates from manufacturing processes and decommissioned components at the end of their service life. In terms of composition, carbon fibers (CF) and CFRP are primarily derived from the automotive and aerospace sectors, as well as manufacturing offcuts, whereas GFRP and glass fibers (GF) predominate in the waste stream of decommissioned wind turbine blades (WTBs) [9,16,17]. Among these waste streams, GFRP waste generated from decommissioned WTBs is not only enormous in quantity but also characterized by a complex multiphase composite structure composed of fiber, polymer matrix, balsa wood, and metal, making it one of the most representative and challenging targets for recycling [18]. Wind power, as a clean energy source, is expanding rapidly worldwide, with a carbon emission intensity of approximately 12 g CO_2_-eq/kWh [19] and a levelized electricity price of 33.5–87.6 EUR/MWh [20]. It is projected that by 2050, the installed wind power capacity in Europe will reach 450 GW, accompanied by approximately 325,000 tons of waste WTBs [21]. By 2024, China’s installed wind power capacity had reached 446.65 GW, of which approximately 71% of the national wind-generated electricity originated from the Northern, Northwestern, and Northeastern territories [22]. The rapid expansion of wind power infrastructure is triggering a large wave of blade decommissioning, leading to the accumulation of massive quantities of non-degradable solid waste. Therefore, under the combined influence of policy support and accelerated industrial development, achieving the sustainable management of FRP waste, including wind turbine blades, has become an urgent global challenge [23].

Traditional disposal methods for FRP waste, such as landfilling and incineration, are increasingly unable to meet the requirements of sustainable development due to tightening environmental regulations and their low resource recovery efficiency. Landfilling not only occupies valuable land resources but also poses long-term environmental risks, whereas incineration releases hazardous substances such as polycyclic aromatic hydrocarbons (PAHs) and fails to recover high-value fibers [26,27]. Against this background, the development of effective resource-oriented recycling pathways has become imperative. Among these, cement composites are regarded as a key engineering platform for the large-scale utilization of recycled FRP, owing to their high tolerance for variations in feedstock morphology and performance [28,29,30,31,32,33]. This strategy not only helps alleviate environmental burdens but also enables the partial substitution of natural aggregates and cement, thereby directly contributing to the achievement of global carbon reduction goals.

In addition to the challenges associated with the production, recycling, and degradation of GF and GFRP, the growing market demand for lightweight and high-strength materials has also continuously driven the expansion of CF production [10]. Owing to the high energy consumption and high cost of virgin CF (vCF) production, CFRP has become the recycling target with the highest unit value, and the economics of its recycling are closely dependent on the property retention of recycled fibers. The global carbon fiber market is currently valued at approximately USD 7.1 billion [9], and is projected to increase to USD 23.2 billion by 2033 [34,35]. However, the production of vCFs is highly energy-intensive, requiring approximately 183–286 MJ/kg [36], which results in the persistently high cost of CFRP manufactured from vCF, typically in the range of 30–40 EUR/kg, whereas recycled carbon fibers (rCFs) products generally cost only 10–20 EUR/kg. Nevertheless, the current recycling rate of CFRP remains only about 13% [17]. From the perspective of overall waste stream characteristics, GFRP and CFRP represent, respectively, the two most typical systems in terms of largest waste volume and highest unit value and accordingly exhibit substantial differences in recycling strategies and technological priorities. For CFRP in particular, its high energy consumption and high cost mean that the economic and environmental viability of its recycling is fundamentally determined by the property retention of recycled fibers and the value-added generated throughout the entire recycling chain.

Although substantial progress has been achieved in the research of FRP recycling technologies, a systematic comparison and comprehensive evaluation of different recycling routes in terms of material property retention, engineering applicability, and environmental-economic performance are still lacking. Therefore, this paper reviews the existing recycling technologies for FRP waste, evaluates the effects of recycled FRP on the performance of polymer-based and cementitious composites, and examines the balance and optimization pathways among solid waste utilization, recycling value, and product performance, together with their associated environmental and economic benefits. Finally, the key challenges currently facing this field are summarized, and future research directions are proposed to provide a systematic reference for promoting the large-scale engineering application of recycled FRP materials and components.

## 2. Bibliometric Assessment and Regional Characteristics of FRP Waste Generation

**Figure 2:**
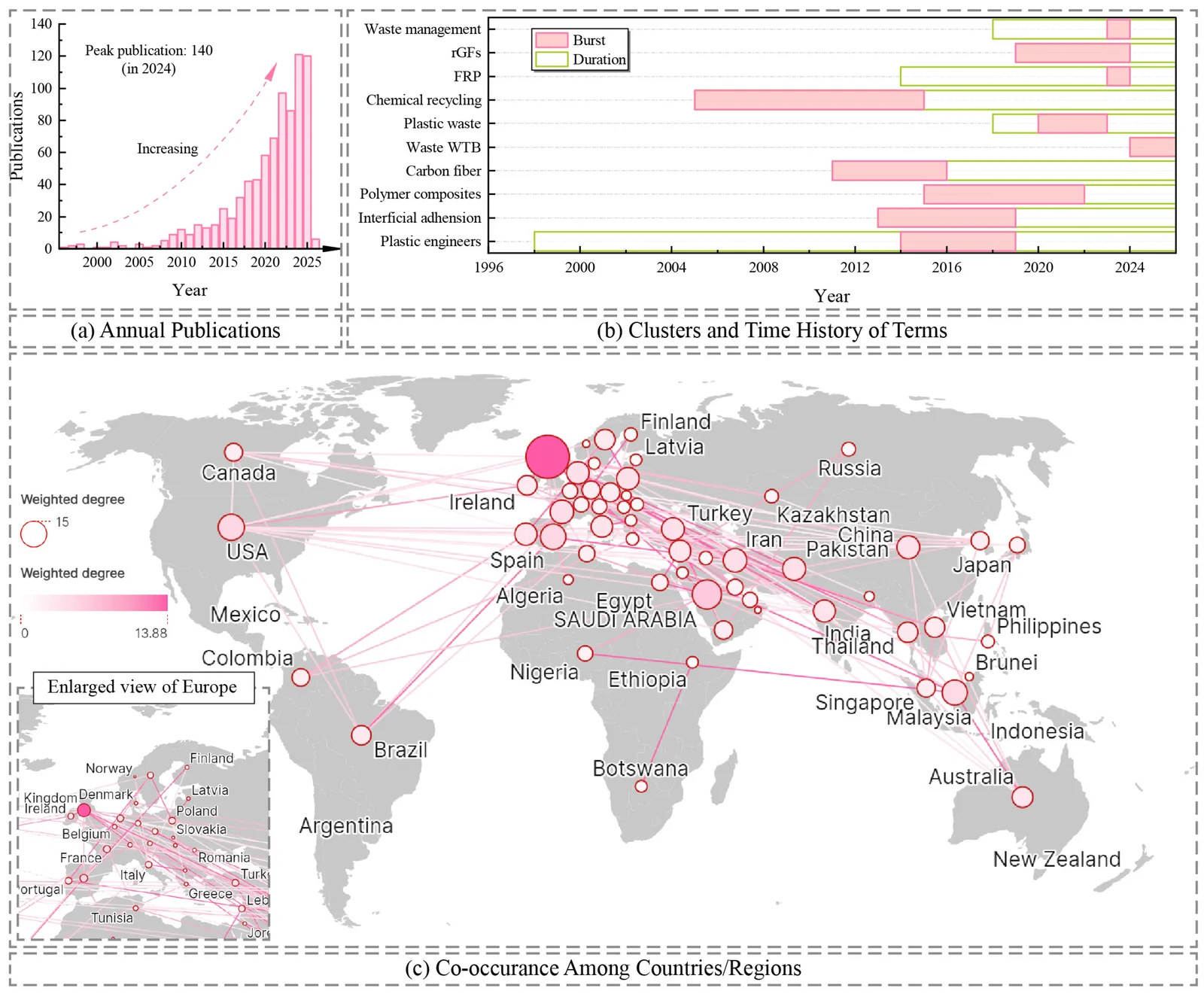
Results of bibliometric assessments: (**a**) annual publications; (**b**) cluster and time history of terms; (**c**) co-occurance among countries and regions.

The recycling and sustainable management of FRP waste have attracted growing research attention in recent years. To systematically map the research evolution of this field, a bibliometric analysis was performed to examine publication trends, keyword co-occurrence patterns, and international collaboration networks [37], as illustrated in Figure 2.

To acquire the information on publications included in Web of Science Core Collection, the title words ((FRP or fiber reinforced polymer or fiber reinforced plastic) or (wind turbine)) and (recycle or waste) were queried without time restrictions. Noticed that the wind turbine is also a critical source of FRP waste. After manually filtering and automatically removing duplicates, 816 publications from 1996 to 2026 (March) were retrieved.

As shown in Figure 2a, the annual number of publications has risen significantly since 2019, reflecting growing research activity. Term clustering and burst detection analysis shown in Figure 2b reveal that studies focusing on GFRP waste management were predominant between 2022 and 2026. Within this, since 2024, attention to the disposal of waste WTB, i.e., the primary source of GFRP, has emerged as a growing research focus, with the growth of wind energy. The national collaboration network presented in Figure 2c demonstrates that Saudi Arabia, the U.K., and the U.S. are the most active contributors and collaborators in this domain, serving as key drivers of its scientific progress.

The geographical distribution of research activity shown in Figure 2c is accompanied by pronounced regional differences in the production, application, and subsequent waste generation of FRP composites. Available regional statistics further illustrate these contrasts.

- Europe: in 2025, thermoset composite waste generation was estimated at about 914 kt, whereas only around 228 kt, or 25%, was considered accessible for recycling, and no more than 5% of this accessible stream was actually recycled [38]. The waste flow spans electronics, transportation, manufacturing scrap, marine products, construction, energy infrastructure, tanks and pipes, consumer goods, and aerospace. Europe therefore exhibits a diversified FRP waste stream, in which collection and retrieval increasingly determine the effective supply of recyclable materials.
- North America: more than 1.9 billion lb of glass-reinforced polyester/vinyl-ester thermoset composites were sold in the U.S. and Canada during the first half of 2025. Construction, infrastructure, and transportation accounted for 34%, 26%, and 21% of sales, respectively [39]. Thus, current composite consumption in North America is strongly concentrated in construction, infrastructure, and transportation applications.
- Asia-Pacific (excluding China): Available records indicate that FRP waste generation in this region is strongly associated with long-service-life products. In Japan, the Japan Reinforced Plastics Society estimates waste FRP generation from historical shipments and product-specific replacement periods, which are approximately 12 years for bathtubs, 15 years for tanks, and 20 years for boats [40]. In New Zealand, more than 1000 t of glass-reinforced composites were used in the Central Interceptor infrastructure project [41]. These records highlight the importance of product replacement cycles and long-lived infrastructure in determining when FRP materials enter regional EoL streams.
- China: production of GFRP reached about 8.89 Mt in 2025, an increase of 18.5% from 2024. This included 4.51 Mt of thermoset and 4.38 Mt of thermoplastic composites [42]. These data indicate the large production scale and continued expansion of both thermoset and thermoplastic GFRP products in China.
- South America: using Brazil as a representative case, the national wind sector is projected to generate approximately 6.25 Mt of total onshore wind-turbine waste by 2050, with 90.61% concentrated in the Northeast region [43]. Although this estimate covers the entire turbine rather than FRP alone, blades are identified as the principal composite component presenting recycling difficulties [43]. Meanwhile, CFRP manufacturing waste from automotive and aerospace companies in Brazil has already been investigated for mechanical recycling and reintroduction into new composite products [44]. These records demonstrate the coexistence of EoL composite components from energy infrastructure and pre-consumer CFRP residues from advanced manufacturing.

These regional differences indicate that FRP waste generation is jointly shaped by industrial structure, product lifetime, in-use accumulation, and waste-management capacity. Taken together, the bibliometric results and regional waste characteristics reveal that the development of FRP waste research is closely associated with the evolution of the global composite industry and the progressive emergence of EoL material streams. Therefore, the subsequent discussion focuses on recycling and reutilization pathways for these heterogeneous waste streams.

## 3. Advances in FRP Waste Recycling and Cementitious Material Applications

### 3.1. Recycling Technologies of FRP Waste

The excellent structural integrity between the polymer matrix and fibers in FRP, together with the inherent durability of FRP itself, has enabled its widespread application across diverse fields, but has also posed substantial challenges for degradation and recycling at the EoL stage. The primary objective of FRP waste recycling is to achieve separation of the fibers from the polymer matrix, or to convert the composite as a whole into reusable forms, such as particles, fibers, or shards. However, the dense cross-linked network of the polymer chains fundamentally constrains the efficiency and economic viability of these recycling processes. At present, the recycling strategies mainly include mechanical, chemical, and thermal recycling, each exhibiting distinct characteristics in terms of technology readiness level (TRL), recycling efficiency, and applicability. Table 1 summarizes reported industrial and pilot-scale applications of these recycling technologies, together with available operational information.

#### 3.1.1. Mechanical Recycling

Mechanical shredding and crushing are currently the most widely applied technologies for FRP waste recycling. It processes waste FRP into blocks, short fibers, or particles of different sizes through physical operations such as cutting, crushing, and grinding. The rFRPs obtained can be either directly utilized or used as pretreatment feedstock for subsequent chemical or thermal recycling processes [32]. Owing to its significant advantages of technological maturity [61], low capital cost [62,63], and ease of large-scale implementation [64,65], mechanical recycling has become the dominant route for the recycling or pretreatment of GFRP in current commercial practice, and also constitutes an indispensable pretreatment step in deep recycling chains. Meanwhile, in some cases, the presence of residual matrix increases the surface roughness of recycled fibers, thereby enhancing the interlocking effect between recycled fibers and the matrix surface, and ultimately improving the tensile-related properties of composite systems [66,67].

However, mechanical recycling is subject to two inherent limitations, namely significant degradation of material properties and the difficulty of completely separating fibers from the matrix [12]. Although post-processes such as electrostatic [68], air-flow [69], or water vibration stratification can further remove the powder fraction [29], residual matrix adhesion remains prevalent on the surface of recycled fibers, indicating that complete fiber–matrix separation is difficult to achieve using mechanical methods alone [70]. Furthermore, for recycled polymer-based composites, pure recycled fibers can more directly perform their bridging effect, whereas the residual matrix on recycled fibers weakens the contribution of fibers to stiffness and strength [71]. By contrast, cementitious composites exhibit better compatibility with the morphological discreteness of reinforcement, residual matrix, and performance fluctuations. In some cases, the roughened surface of recycled fibers and matrix adhesion may even improve interfacial interlocking and crack-bridging effects. Therefore, recycled fibers obtained through mechanical recycling may be less suitable for direct reuse in polymer-based composites, whereas they offer promising opportunities for resource utilization in cement composites [72,73,74].

#### 3.1.2. Chemical and Electro-Chemical Methods

To overcome the limitations of mechanical recycling strategies and thereby enable the value-added reutilization of waste CFRP, chemical and electro-chemical strategies have attracted increasing attention as key alternatives capable of preserving fiber integrity. These methods selectively degrade or decompose the polymer matrix through chemical approaches, such as solvolysis and electrolysis, while maximizing the retention of fiber morphology and mechanical properties [75,76,77,78]. They may also convert FRP wastes into higher-value functional products [79,80]. As summarized for the chemical and electrochemical routes in Table A1, the effectiveness of these processes is jointly governed by solvent or electrolyte composition, catalyst type, pressure, temperature, and the structure of the target polymer matrix, resulting in substantial variations among studies and making direct cross-comparison difficult.

Solvolysis separates fibers from the matrix by selectively cleaving polymer chains under controlled temperature and pressure. By avoiding high-temperature combustion, this method enables matrix removal while better preserving the morphology and mechanical properties of the fibers [11,81,82,83,84]. Moreover, under supercritical ethanol treatment at 275 °C with 1 wt.% NaOH for 40 min; a resin-removal rate of 97.9% was achieved, while the tensile-strength retention ratios of rGFs and rCFs reached 95.64% and 97.79%, respectively [77]. Currently, research on solvolysis is shifting from mere matrix removal toward the value-added recovery of all components. For example, the introduction of Lewis acids or transition-metal catalysts can enable efficient hydrolytic and mild oxidative degradation of epoxy networks, thereby significantly reducing chemical damage to the fiber surface [85,86]. Meanwhile, the use of moderate-temperature solvent systems combined with catalysts can partially depolymerize, thus simplifying subsequent separation and purification processes [87,88,89]. For specific matrix systems, organic solvent systems may even enable multiple closed-loop recycling of both matrices and fibers [90,91]. Despite the clear advantages in terms of recycling quality and material circularity, the engineering-scale application of solvolysis remains constrained by several factors, including high solvent cost, long processing time, harsh reaction conditions, and relatively low TRL [61,92]. Therefore, these solvent-based methods are currently regarded primarily as a preferred route for high-value recycling or for specific waste streams, rather than as a universal solution for the large-scale treatment of mixed FRP waste.

Electrochemical recycling is a technique that operates under atmospheric pressure and mild temperatures (typically below 100 °C), in which an externally applied electric field drives electrode reactions to matrix selective decomposition or to achieve functional surface modification of the material. This technique exhibits notable advantages in terms of mild reaction conditions and precise process controllability [5,76]. It can efficiently preserve the mechanical properties of the fibers, with tensile strength retention generally exceeding 80% [76,93,94], while also allowing the electrolyte to be reused, thereby offering favorable process economics and environmental compatibility [95]. In addition to serving as a major route for fiber separation and recovery, this method can also function as a key aftertreatment strategy for recycled CFRP. Through controlled electrochemical surface oxidation, oxygen-containing functional groups can be precisely and selectively introduced, thereby effectively enhancing the interfacial bonding performance between recycled fibers and cement-based or alkali-activated materials [56]. Moreover, it enables the directed conversion of waste FRP into high-value functional materials, such as graphene oxide with photothermal responsiveness [79] or structural ceramic SiC [80], thus substantially upgrading the application level and market value of the recycled products. Owing to its combined advantages in fiber property retention, resource circularity, and high-value product conversion, electrochemical recycling has emerged as one of the key development directions for achieving high-value and resource-efficient recycling of composite waste.

Overall, chemical and electrochemical recycling can preserve fiber quality under comparatively mild conditions, but their efficiency and versatility remain strongly dependent on process design. In chemical systems, the effectiveness of polymer chain cleavage does not depend solely on the solvent. Catalyst composition, solvent-catalyst interactions, reaction intermediates, and treatment sequence may either promote or inhibit matrix decomposition and should therefore be optimized as a coupled reaction system [96].

#### 3.1.3. Pyrolysis and Oxidation Methods

**Figure 3:**
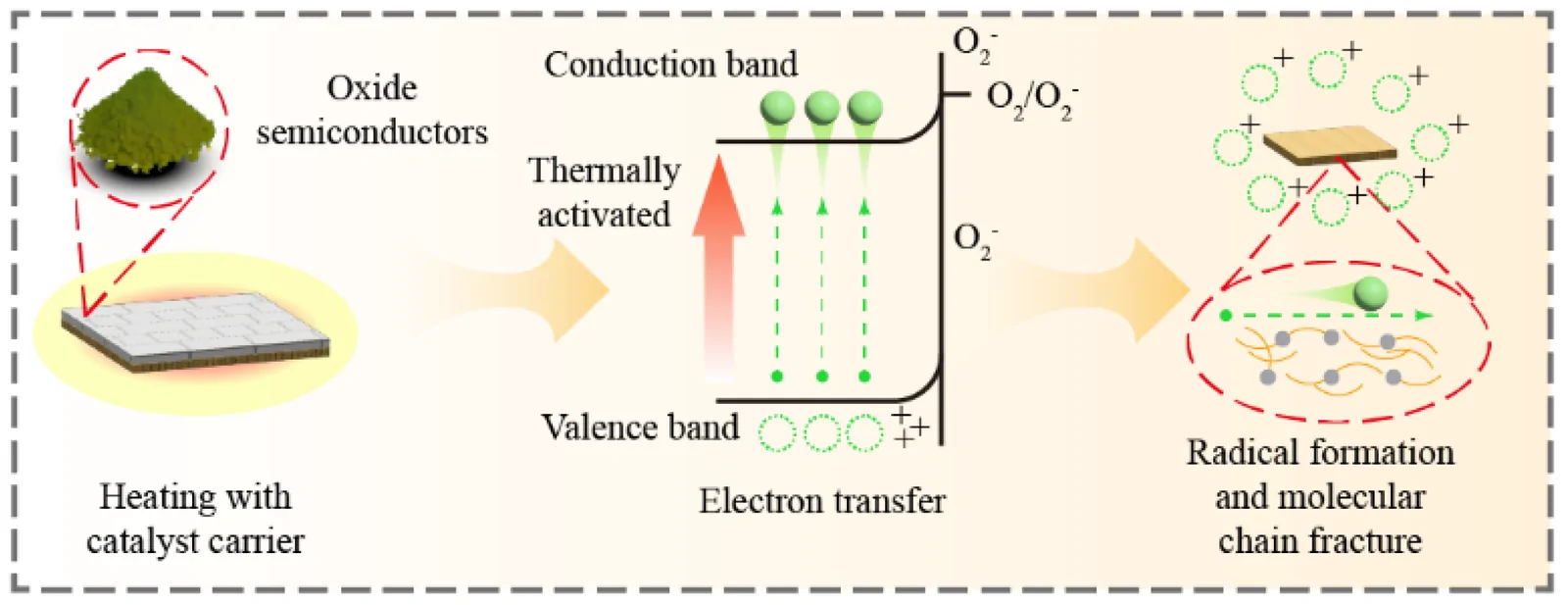
Effect mechanisms of thermally activated semiconductive catalyst [108,110,112].

As one of the few CFRP recycling technologies that have been implemented at an industrial scale, thermal recycling enables effective separation of fibers from the matrix by heating waste FRP to 350–500 °C under anaerobic conditions, thereby decomposing the polymer matrix [27,97,98]. This process mainly includes direct heating, fluidized bed treatment, and microwave pyrolysis [99,100,101,102], and is characterized by relatively high TRL, versatility, large processing capacity, and considerable recycling efficiency [56,57,103]. Although a certain amount of pyrolysis oil or amorphous carbon may remain attached to the surface of the recycled fibers, the crystalline structure and mechanical properties of the fibers themselves remain close to those of virgin fibers [104]. Moreover, the pyrolysis process can optimize the distribution of oxygen-containing functional groups on the surface of recycled fibers, while the roughened surface further enhances their surface activity. This is beneficial for improving fiber–matrix bonding in subsequent FRP remanufacturing processes [105,106]. Despite its industrial applicability, the relatively high energy consumption of this process (22.2–66.3 MJ/kg) remains a major constraint on its further development [84,107]. To address this issue, catalytic-assisted pyrolysis has been developed, in which metal oxide catalysts such as TiO_2_, Cr_2_O_3_, and Fe_2_O_3_ are introduced to promote polymer bond cleavage at lower temperatures (Figure 3) and effectively suppress the generation of harmful gases such as CO and alkanes [108,109,110,111,112]. In addition, these catalysts can be reused [113], while catalyst-assisted treatment can substantially reduce the thermal energy required for fiber separation by lowering the required treatment temperature and duration [114].

While reducing energy consumption, the recovery of fiber performance and interface optimization become other key factors. The pyrolysis residue weakens the interface bonding with the resin and typically requires oxidative post-treatment to remove it, thus exposing the intact fiber structure [115,116,117]. Compared to the random roughness increase caused by the untreated residual matrix, the pyrolysis-oxidation treatment results in better mechanical performance stability [66]. In terms of oxidation process optimization, machine learning models are now available to predict the microstructure and mechanical properties of recycled fibers based on process parameters, guiding process control [115].

**Figure 4:**
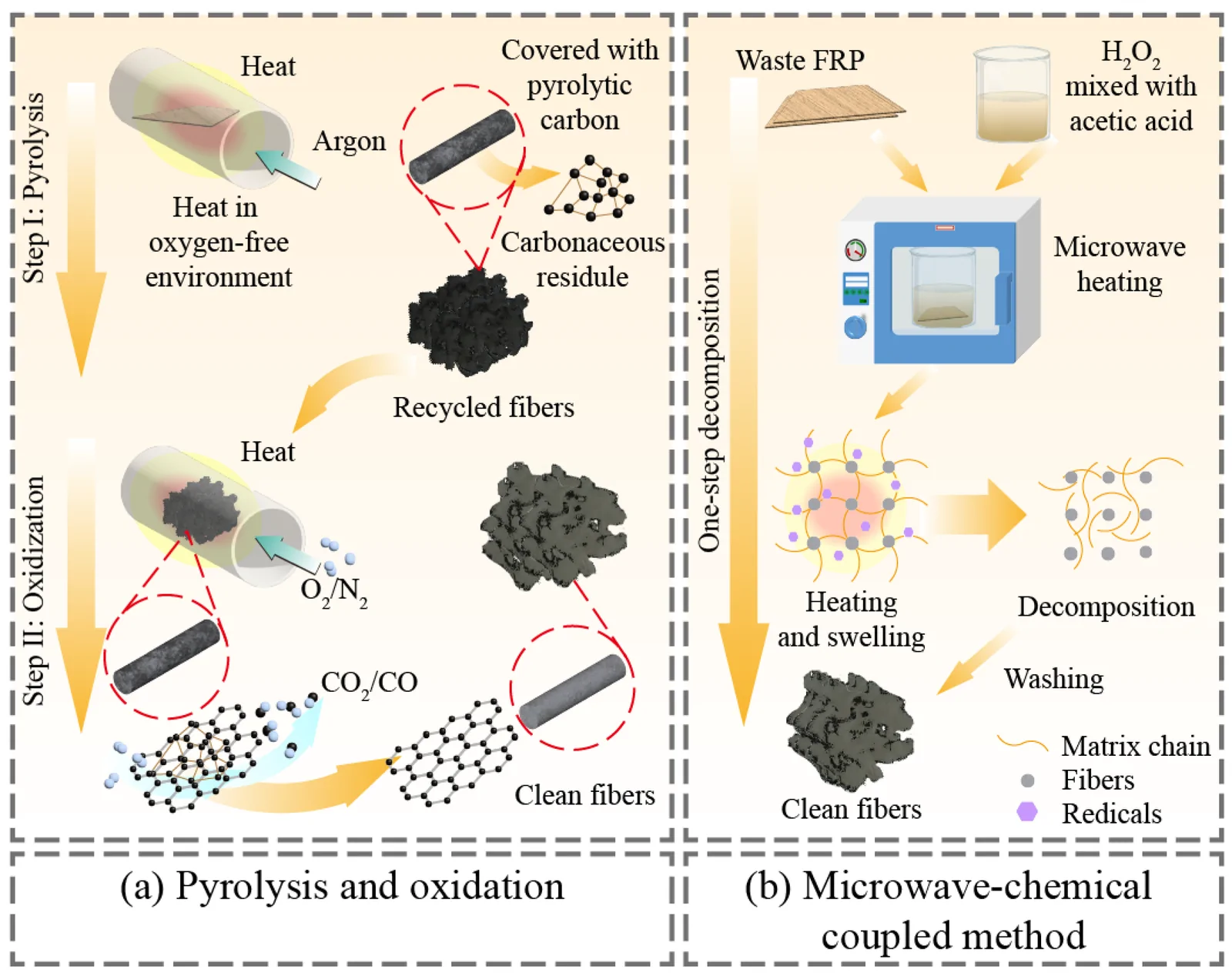
Workflow of thermal recycling methods: (**a**) pyrolysis and oxidation and (**b**) microwave-assisted thermochemical recycling.

Compared with fluidized bed and conventional pyrolysis methods, microwave recycling can induce matrix pyrolysis through microwave irradiation at a lower energy consumption of approximately 10 MJ/kg [84]. The main advantages of this method lie in its high energy efficiency, process flexibility, and ease of control [87,102]. In addition, microwave pyrolysis exhibits unique potential in thermo-chemical coupled pathways, particularly in terms of low-temperature processing, reduced fiber damage, and the elimination of complex post-treatment procedures, as shown in Figure 4. For instance, under microwave irradiation, hydroxyl radicals generated from H_2_O_2_ and acetic acid can preferentially attack weak bonds in the epoxy chains, such as C-N bonds, thereby enabling controlled matrix depolymerization while causing minimal damage to the fibers. Reported by Rani et al. [118], after 30 min of H_2_O_2_/acetic-acid pretreatment at room temperature, microwave-assisted treatment at 700 W for a total exposure time of 180 s retained approximately 99% of the tensile strength and more than 90% of the elastic modulus of rGFs. The resulting GFRP laminates exhibited mechanical properties comparable to, or even superior to, those of the virgin counterparts [67].

In summary, thermal recycling should be regarded as an integrated system composed of the core pyrolysis process, catalytic enhancement, and oxidative aftertreatment. Although it remains constrained by relatively high energy consumption and the need for aftertreatment, its comparatively high industrial maturity and processing efficiency make it more engineeringly feasible than most chemical/electrochemical methods in large-scale, high-value recycling scenarios where fiber property retention is a primary objective. Future advances in catalyst optimization, intelligent process control, and the further development of thermo-chemical coupled technologies are expected to further reduce energy consumption while improving the quality of recycled fibers.

#### 3.1.4. Innovative Strategies in FRP Recycling

To achieve a compromise among fiber property retention, operational simplicity, cost-energy efficiency, and versatility, recent research has gradually shifted from forceful disintegration toward interfacial weakening-based recycling strategies derived from the in-service damage mechanisms of FRP [119]. Rather than relying on complete decomposition, the principal concept of this approach is to selectively weaken the matrix-fiber interfacial transition zone (ITZ) and interlaminar bonding strength [120], thereby directing the material to undergo controlled disassembly along its weak ITZ. In this way, the integrity of the fibers can be preserved to the greatest extent possible while enhancing the value of the recycled products. The damage evolution of FRP generally initiates from matrix cracking and interfacial debonding, with the ITZ serving as the weakest link due to its lower bonding energy and greater environmental sensitivity [119]. Based on this principle, interlaminar weakening methods actively accelerate interface degradation and induce controlled delamination by applying external physical fields, such as high-voltage fragmentation [121,122,123], ultrasonic vibration [124,125,126], rapid freeze–thaw cycling [127,128], nanosecond pulsed laser [129], or hygrothermal coupling [130].

Quantitative evidence supports interface-targeted disassembly. After 10 days of freeze–thaw treatment, internal crack volume and connected porosity increased by about 65% and 32%, while embedded glass fibers retained 93–96% of their nano-mechanical properties [127,128]; while under the optimized two-step laser treatment at a +130 mm defocus distance and step powers of 420/120 W, the resin removal rate reached 97.92%, while rCF retained 93.38% of its tensile strength and 105.77% of its tensile modulus. The total processing time was reduced from 309 to 147 s relative to the unoptimized process, corresponding to a 110.20% increase in recovery efficiency [129]. However, freeze–thaw cycling remains slow, whereas laser processing requires strict control to avoid localized thermal damage. These innovative recycling methods have demonstrated potential advantages under laboratory conditions in terms of process controllability and preservation of fiber integrity; however, their processing efficiency, energy and economic performance, and feasibility for large-scale engineering implementation still require more systematic evaluation and validation.

### 3.2. Recovered-Fiber Quality and Comparative Assessment of Recycling Technologies

#### 3.2.1. Quality Retention of Recovered Fibers

**Figure 5:**
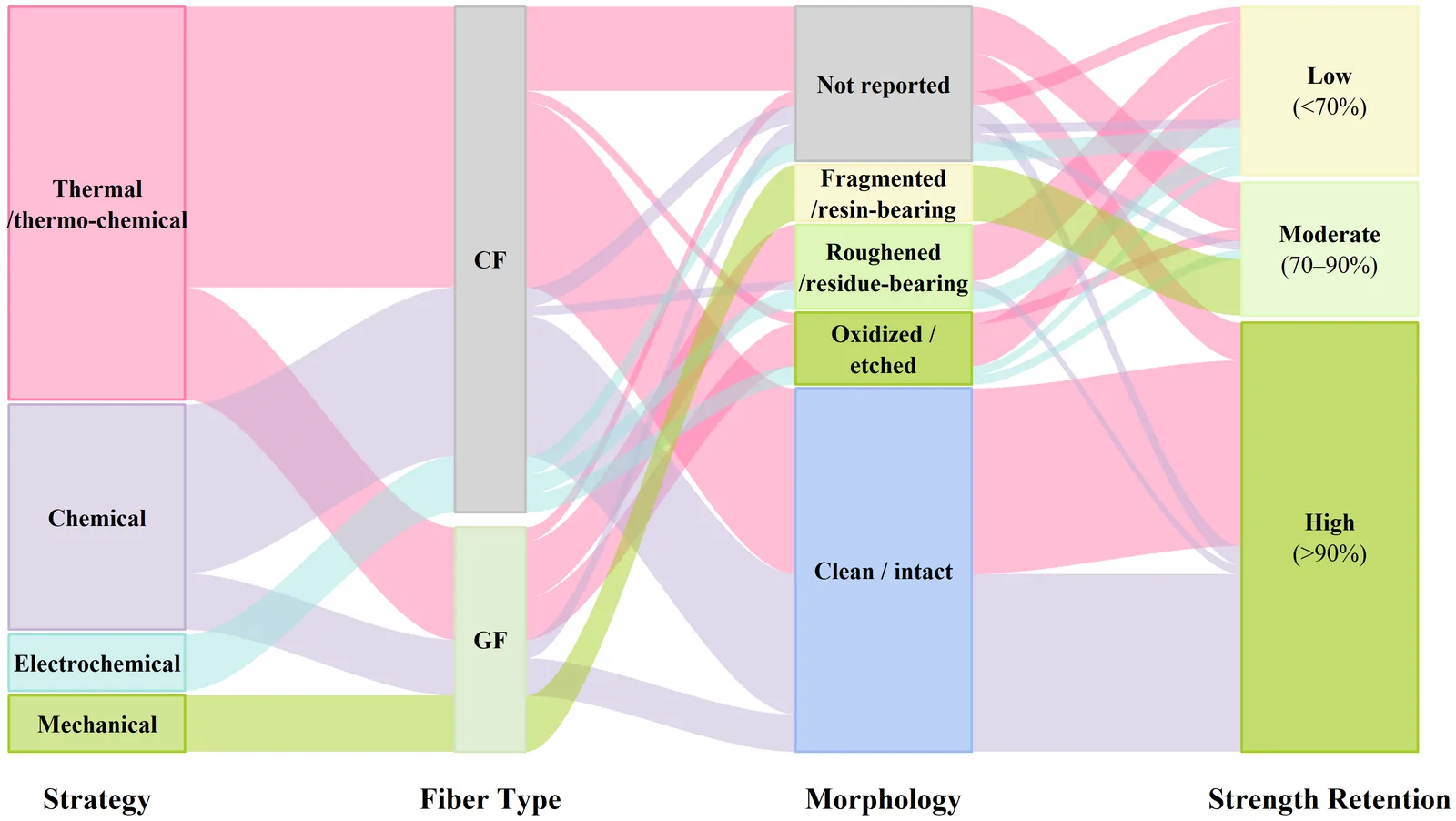
Distribution of recovered-fiber morphology and strength retention across recycling strategies and fiber types.

Table A1 summarizes the recovered forms, surface and structural conditions, and mechanical property retention of rGFs and rCFs obtained using different recycling strategies. Such emerging techniques, e.g., freeze–thaw cycling and pulsed-laser treatment, were excluded from discussion because the available reported data remain limited currently. Based on Table A1, Figure 5 classifies the existing studies according to recycling strategy, fiber type, morphological condition, and strength retention level, thereby illustrating the distribution of recovered fiber quality associated with different processes and fiber types. In Figure 5, the width of each flow is proportional to the number of literature entries assigned to the corresponding connection. Recovered fiber quality cannot be evaluated solely by matrix removal efficiency, but should also consider fiber continuity, residual resin or char, surface roughening and etching, and mechanical property retention. The available data are mainly concentrated on thermal, thermo-chemical coupled, and chemical recycling, with CF being the principal target. The rCFs recovered through these routes are generally characterized by clean surfaces or by etching and oxidation traces, while retaining a high proportion of their original strength (≥90%). By contrast, the studies on mechanical recycling mainly concern GF, and the recovered products are typically fragmented or resin-bearing, with moderate strength retention of 70–90%.

Mechanical recycling primarily causes quality loss through surface damage induced by crushing and grinding. These processes convert continuous reinforcements into short filaments, fiber bundles, and resin-coated fragments, while introducing macro- and mesoscopic defects such as broken ends, longitudinal scratches, and local splitting on the fiber surface, thereby degrading the mechanical performance of the recovered products. For example, E-GFs recovered from an epoxy matrix exhibited reductions of 19.1–19.9% in tensile strength and 25.0–33.7% in rupture strain. However, because mechanical cutting and grinding do not alter the glass-network structure of rGF, an insignificant reduction in elastic modulus was observed [66]. Residual matrix attached to the fiber surface also increases the morphological variability of the recovered products and alters their wettability and interfacial bonding with the new matrix.

Chemical and electrochemical recycling can effectively remove the polymer matrix from the fiber surface, but the recovered fiber quality depends on the compatibility between the reaction medium and the FRP type. For example, a supercritical ethanol/NaOH system achieved a matrix-removal efficiency of 97.9% at 275 °C, 1.0 wt.% NaOH, and 40 min. However, when the treatment temperature increased to 325 °C, the tensile-strength retention of rGF decreased to 30.40%, whereas that of rCF remained at 99.24%. This indicates that high-temperature alkaline media can attack the glass network and generate surface defects, while CF exhibits greater chemical stability in the same system [77]. After 21 d of electrochemical treatment, the maximum tensile-strength retention of rCF reached approximately 80% at 3% NaCl and 25 mA, but increasing the NaCl concentration to 20% reduced the corresponding retention to 32%, 43%, and 65% at applied currents of 4, 20, and 25 mA, respectively [76]. Therefore, chemical and electrochemical recycling processes should be selected and optimized according to the compatibility between the reaction system and the fiber type.

Strength degradation during thermal treatment mainly results from sizing decomposition, surface defect propagation, and subsequent oxidative ablation. The quality of thermally recovered fibers is therefore governed by the pyrolysis temperature, reaction atmosphere, and post-treatment intensity. For example, flue-gas pyrolysis at 420–450 °C for 5–6 h achieved a matrix removal efficiency above 99%, with a tensile strength loss below 10%; however, a further increase in temperature improved fiber purity while intensifying fiber damage [131]. For CFRP, a two-step process consisting of pyrolysis at 600 °C for 30 min followed by oxidation at 450 °C for 15–20 min retained more than 90.19% of the tensile strength and 84.44% of the elastic modulus of vCFs [132]. These results indicate that matrix removal and fiber-property retention should be jointly optimized. The upper temperature limit and oxidation duration should be controlled while ensuring sufficient resin decomposition and char removal, thereby avoiding fiber damage caused by excessive oxidation. Thermo-chemical coupled recycling uses solvent pretreatment to swell the resin and partially cleave its molecular chains, thereby reducing the thermal input required for subsequent pyrolysis. With acetic-acid solvolysis pretreatment at 100 °C without isothermal dwelling, followed by pyrolysis up to 425 °C and oxidation up to 550 °C at 15 °C/min, the tensile strength retention of rCF reached 90.53% [81]. The key role of thermo-chemical coupling is therefore to reduce the thermal stability of the resin network through pretreatment, allowing subsequent pyrolysis to proceed at a lower temperature and under milder oxidation conditions, while simultaneously reducing energy consumption, char formation, and fiber damage. However, solvent recovery and waste liquid treatment must consequently be incorporated into the process design and optimization.

Overall, recovered-fiber quality is jointly controlled by the FRP type, recycling system, and treatment process. Mechanical recycling mainly changes fiber length, integrity, and surface condition and generally does not directly damage the glass-network structure of GF; therefore, its elastic modulus can be largely retained. However, broken ends, scratches, and local splitting induced by cutting and grinding reduce fiber strength, while residual matrix on the fiber surface also affects subsequent wetting and fiber–matrix bonding. Thermal, thermo-chemical coupled, and chemical recycling can achieve high strength retention for both GF and CF under suitable media and treatment conditions, although the sensitivities of the two fiber types to temperature and reaction media differ substantially. GF is more susceptible to surface attack in high-temperature alkaline media. Thermal recycling generally provides better property retention for CF, whereas GF is more vulnerable to sizing decomposition, thermally induced defect propagation, and oxidative damage.

#### 3.2.2. Comparisons and Challenges in Recycling Technologies

The development and application prospects of FRP waste recycling strategies are essentially governed by the complex trade-offs among recycling cost, energy consumption, environmental impact, and TRL [133,134,135,136] (Table 2). At present, different recycling pathways exhibit substantial variations in both cost and environmental performance, which directly determine their commercialization potential and the boundaries of their applicable scenarios. The comparison should additionally consider the quality and form of the recovered products, as well as their compatibility with the intended end-use application. The central challenge facing current recycling strategies therefore lies in achieving a coordinated balance among recycling efficiency, energy consumption, economic cost, fiber property retention, and the utilization value of the recovered products.

Specifically, mechanical recycling strategies are characterized by high technological maturity, low cost, and broad applicability, and therefore occupy a dominant position in industrial practice; however, the recycled products are mostly limited to downcycled applications, making it difficult to effectively preserve the structural value of the fibers [137,138]. Nevertheless, their relatively high processing efficiency and tolerance to heterogeneous waste make them more suitable for large-scale disposal and valorization, such as reutilization in cementitious composites.

For rCFRP, the environmental value of recycling depends mainly on whether the recovered fibers retain sufficient quality to replace vCFs. High-quality rCFs can compensate for part of the environmental burden of energy-intensive recycling when effective substitution is achieved [139,140,141]. Cutting and shredding may shorten or damage the fibers, reduce their substitution potential, and confine the recovered products to low-value applications. The rCF recovery yield determines the amount of vCF displaced, while plant capacity affects the energy and capital burdens assigned to each unit of rCF. Chemical and electrochemical methods can retain fiber properties under relatively mild conditions, but their wider application is limited by processing cost, energy demand, and scalability. Thermal recycling has achieved a higher level of industrialization and can preserve fiber structure, although its high-temperature operation remains energy intensive. These methods are therefore most suitable for high-value CFRP waste, where the retained fiber quality and the substitution of vCFs can justify the additional processing requirements.

Chemical and electrochemical methods can retain fiber properties under relatively mild conditions, although their application remains limited by cost, energy demand, and processing scale [76]. Thermal recycling has achieved greater industrial maturity and can recover fibers with relatively high structural integrity, but high-temperature operation and subsequent treatment increase its energy demand [47,142]. Interfacial and interlaminar weakening methods and thermo-chemical coupled strategies also show potential by using mild temperatures and improving fiber property retention. Their processing efficiency, environmental and economic performance, and industrial scalability still require further validation. Moreover, this strategy should also intervene at the material design and product stage. Incorporating selectively cleavable groups into thermosetting networks can provide high-performance FRP with predetermined chemical disassembly pathways, reducing the severity of fiber-recovery conditions and facilitating the recovery of both fibers and matrix-derived products [91,143].

In addition, high-value CFs and their waste should be recovered with maximum retention of their structure and properties for composite remanufacturing, whereas large-volume GFRP waste is better suited to mechanical processing for use as construction reinforcements or aggregates. Heterogeneous waste should be classified and allocated according to product quality. Such differentiated strategies can better balance environmental benefits, economic feasibility, technical maturity, and application demands.

**Table 2:** Comparative analysis of recycling techniques across multiple metrics.

Method	Landfill & Inceneration	Mechanical Recycling	Freeze–Thaw Cycling [127,128]	Chemical & Electrochemical Recycling	Thermal Recycling
Reaction temperature	More than 400 °C (incineration)	-	Sub-zero	Less than 200 °C for a low-temperature system.	400–700 °C
Fiber retention	None	70–82% of tensile strength [144] 98% of elastic modulus [104].	96% of the elastic modulus	More than 90 of tensile strength [76,93]	80–99% of tensile strength [115,116,118]
Energy use per kg of waste treated	0.4 MJ (landfilling) [145] −4.2 MJ (incineration) [138]	0.11–0.27 MJ [146,147]	-	49.8–66.3 MJ (Solvolysis) [148]	10–66.3 MJ [84,107,149]
Efficiency	High	High	Low	Low (Solvolysis and electrochemical)	Medium
TRL ^1^	Commercial (9)	Commercial (8–9)	Laboratory (3)	Laboratory or pilot (3–6)	Pilot (5–7)
Cost per kg of waste treated	Low ~0.092 EUR (UK, 2015) [149] ~2.0 EUR (Belgium, 2025) [65]	Low 0.52–5.4 USD [139,150]	-	Depends on the use of reagents 3.99–51.2 EUR (solvolysis) ^2^ [92,148]	Medium ~5.0 EUR [148,149]
Capital	Operation	Equipments	-	Reagent (Solvolysis) Equipments (electrochemical)	Equipments
Environment concern	Microplastic migration, greenhouse gas emissions, and toxic residues	Dust	Low toxicity and microplastic content in water.	Toxic liquid	Toxic and greenhouse gases Dust (fluidized bed)
GWP per kg waste treated	~0.024 kg CO_2_-eq (landfilling) [145] 0.24–3.09 kg CO_2_-eq (incineration) [65,145,151]	~0.22 kg CO_2_-eq [148]	-	0.64–5.58 kg CO_2_-eq (Solvolysis) [92] ~0.08 kg CO_2_-eq (HVF) [148]	1.92–2.88 kg CO_2_-eq [148,152]
Advantages	Simplicity and volume disposal.	High efficiency and low energy demand.	Potential for recovering continuous fibers, with low toxicity and carbon footprint.	High fiber quality recovery.	Robust fiber recovery with moderate strength degradation.
Application	Final processing.	Directly utilized in cement composites.	Extracting high-value fibers.	Extracting high-value fibers.	Extracting high-value fibers.
Challenges and limitations	Permanent resource loss, high greenhouse gas emissions, and environmental externalities.	Fiber property degradation is limited to low-value applications.	Low processing efficiency; limited industrial maturity.	High solvent and reactor cost; complex separation and limited scalability.	High energy demand and capital cost; post-treatment is required for fiber quality recovery.

Note: ^1^ the TRL scores were ranked by 1 to 9 for lab (1–4), pilot (5–7), and commercial (8–9) scales, respectively [84,133,134,135]. ^2.^ 1.0 USD = 0.87 EUR = 95.44 INR, in August 2026.

### 3.3. Direct Reuse in Infrastructures

**Figure 6:**
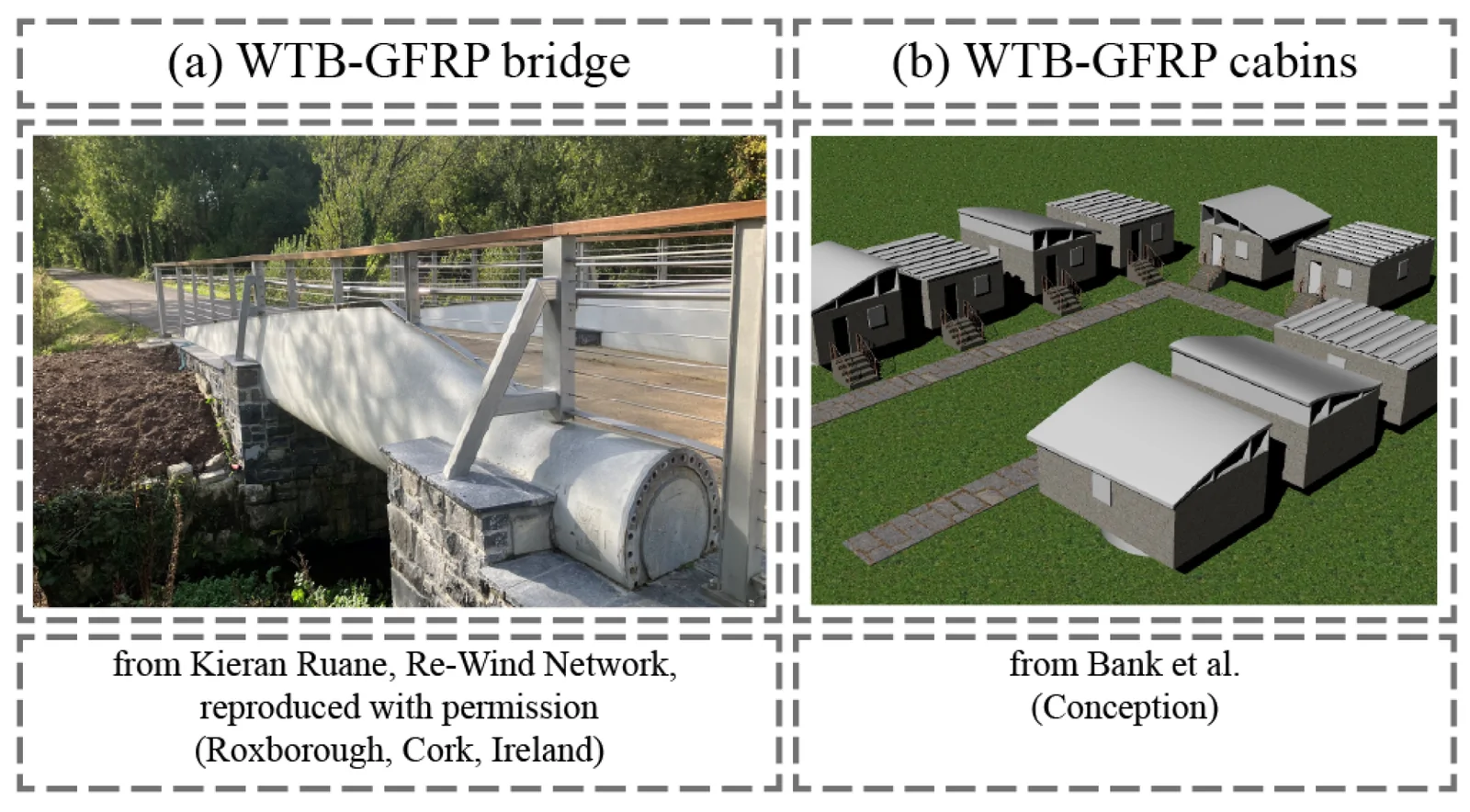
Examples for direct reuse in infrastructures: (**a**) bridge [156] and (**b**) cabins [158].

Direct reuse is a recycling strategy in which discarded FRP components, such as decommissioned GFRP-WTBs, are applied directly in new engineering works after minimal cutting and processing. This approach aims to maximize material value while minimizing energy consumption and cost, thereby providing an effective utilization pathway [153]. Typical applications include the use of such components as transmission towers [154,155], structural elements [156], containers [51], etc., as shown in Figure 6. Owing to the inherently high strength, high stiffness, and excellent durability of GFRP, direct reuse can significantly reduce the consumption of virgin construction materials such as steel and concrete while maintaining structural load-bearing capacity, thereby effectively lowering life-cycle carbon emissions. Without damage from extreme loads, the stiffness of waste GFRP components after long-term service has insignificant degradation, further confirming that classical composite mechanics theory remains applicable to such materials [157], which provides a theoretical basis for their standardized reuse.

The principal costs associated with direct reuse arise from the cutting, transportation, and on-site installation of waste FRP components [159], among which the cutting cost is approximately 0.16 USD/kg, comparable to that of mechanical recycling [160]. Although the cutting process inevitably generates secondary waste, its total amount is substantially lower than the volume of the original FRP waste. Despite the considerable potential of this approach in terms of environmental and economic benefits, its large-scale implementation remains constrained by the geometric characteristics of decommissioned components, site-specific logistical conditions, and the absence of corresponding engineering codes and design standards. Consequently, direct reuse is currently still largely confined to customized and demonstration-scale applications and has not yet developed into a systematic and replicable industrial pathway.

### 3.4. Remanufacturing Routes and Interfacial Control of Regenerated FRP Composites

Remanufacturing recovered fibers into regenerated FRP composites can retain part of the material value of CFRP waste. Its environmental benefit depends on whether rCFs can be converted into products capable of replacing materials manufactured with vCFs [140,161]. Recycling and pretreatment commonly shorten the fibers, broaden their length distribution, remove the original sizing, alter the surface condition, and leave residual matrix or char [36,60,162,163]. These changes affect fiber handling, dispersion, impregnation, and compatibility with the new matrix. Depending on the recovered fiber form, rCFs and rGFs can be processed into nonwoven mats, aligned tapes, recycled laminates, pellets, filaments, or printable feedstocks [51,164,165,166,167,168,169,170].

Fiber reconstruction and orientation strongly affect the reinforcement efficiency of regenerated composites. Wet-laid and hydrodynamic alignment processes can reorganize fluffy and entangled short fibers into compact and directionally reinforced preforms [171]. Remanufactured composites retained much of their performance after the first recycling cycle. However, a second cycle caused fiber shortening and residual-matrix accumulation, followed by lower fiber alignment, fiber volume fraction, stiffness, and strength [172]. It is confirmed that recovered fibers can be incorporated into epoxy panels, recycled laminates, and higher-value composite products when their length, arrangement, and surface condition are adequately controlled [60,113,166,167,168]. Fiber discontinuity still produces ends, overlaps, and resin-rich regions, which reduce stress-transfer efficiency and can initiate local damage [67,168].

The fiber surface determines load transfer across the fiber–matrix interphase. Loss of sizing, residual matrix, surface oxidation, and chemical incompatibility with the new polymer can alter interfacial adhesion. For example, Burn et al. [162] reported that removing the original epoxy-compatible sizing by simulated pyrolysis or solvolysis increased the interfacial shear strength of CF/PP systems by 4% and 33%, respectively. The addition of 2 wt% maleic-anhydride-grafted polypropylene further increased the interfacial shear strength by 240–330%, because its polar anhydride groups interacted with oxygen-containing groups on the carbon-fiber surface, while its polypropylene backbone remained compatible with the PP matrix. Catalytic thermal treatment can also modify the surface chemistry of rCFs. Under the conditions reported by Cheng et al. [113], regenerated CFRP retained 92.88% and 92.51% of the tensile and flexural strengths, respectively. These results show that surface treatment, resizing, and coupling agents should be selected for the specific fiber–matrix combination.

**Figure 7:**
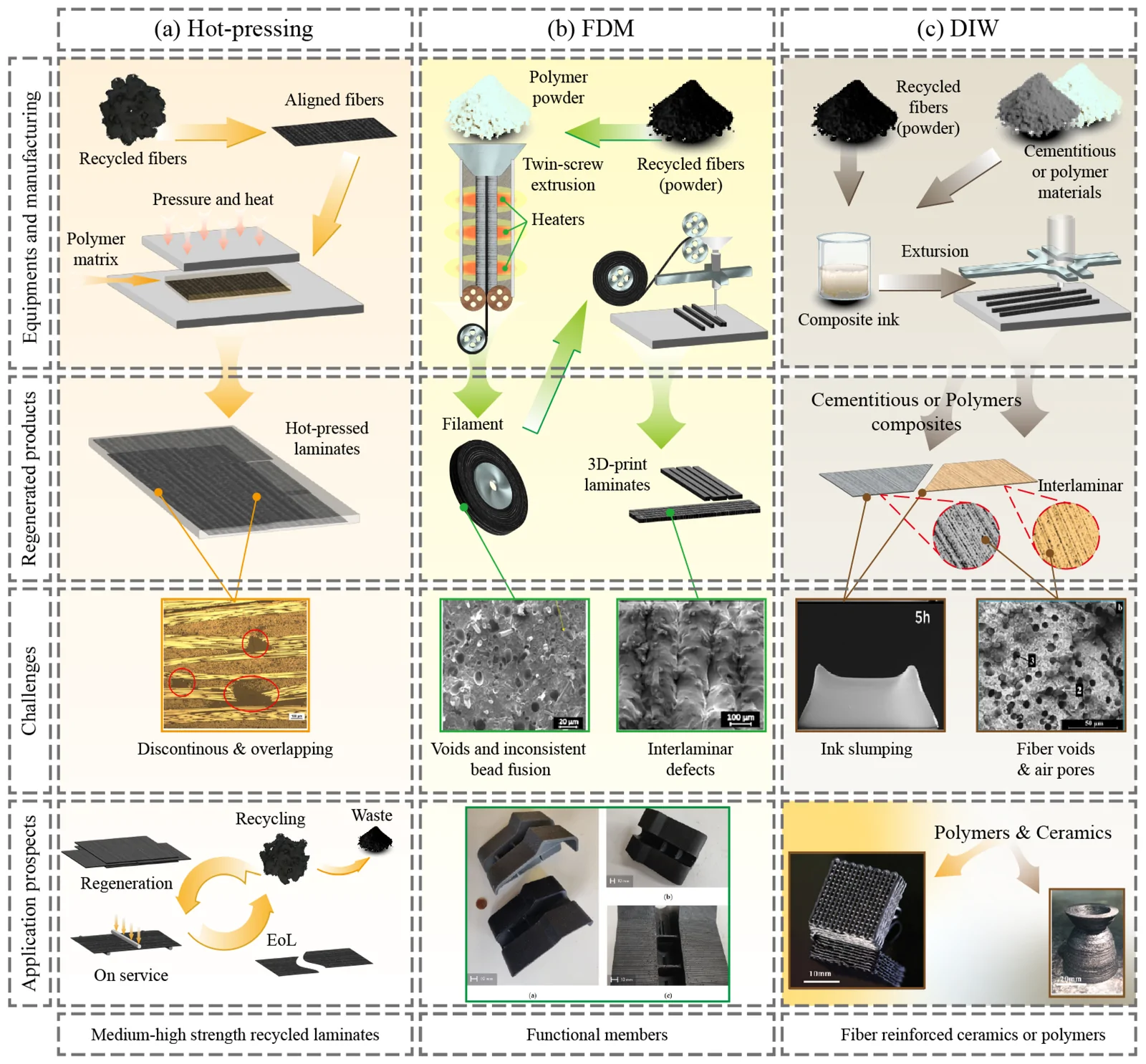
Remanufacturing via (**a**) hot-pressing, (**b**) FDM, and (**c**) DIW: techniques, challenges, and applications of regenerated FRP. (Figure elements sources: [164,191,192,193,194,195]).

As shown in Figure 7, additive manufacturing offers additional processing routes for short-cut or milled fibers and recycled powders that are difficult to form into conventional laminates [17,49,164,165,173,174]. Fused deposition modeling (FDM) requires compounding, pelletization, and filament extrusion before printing [169,175,176]. Fiber breakage may occur during each stage, while fiber content affects melt flow, orientation, impregnation, and porosity [176,177,178]. Direct ink writing (DIW) uses shear-thinning inks containing fibers or powders and allows greater flexibility in feedstock composition [179,180,181]. Its forming quality depends on solids content, ink rheology, fiber dispersion, deposition stability, curing, and dimensional retention [182]. Studies on metallic and ceramic systems also provide useful process information on ink design, deposition, melt infiltration, shrinkage control, and post-treatment [183,184,185,186]. Printed fiber paths and loading rates can further affect the progressive failure of fiber-reinforced components [187]. Both FDM and DIW may introduce voids, incomplete fusion, and weak interlayer or inter-bead regions, which restrict load transfer even when the fibers retain satisfactory mechanical properties [188,189,190].

For FDM, interlayer defects can be reduced by optimizing fiber content and deposition parameters and by applying matrix-compatible thermal post-treatment to improve inter-bead and interlayer fusion [163,196,197,198,199], whereas DIW requires coordinated control of ink rheology, printing path, and curing conditions to maintain shape stability, fiber alignment, and inter-bead and interlayer bonding [180,184]. Nevertheless, the performance of regenerated composites containing discontinuous recycled fibers also depends on recovered-fiber quality, fiber–matrix compatibility, and forming-induced defects. Alignment parameters should therefore be adjusted according to the recovered-fiber length distribution, while resizing and coupling agents should be selected to match the chemistry of the new matrix. Current optimization of regenerated FRP composites, particularly in additive remanufacturing, remains largely focused on processing parameters and post-treatments, whereas the intrinsic variability of recovered fibers, including fiber-length distribution, residual matrix, surface chemistry, and quantitative fiber–matrix load transfer, has received comparatively limited attention. This gap hinders the establishment of transferable relationships among recovered-fiber interfacial properties. Furthermore, life-cycle assessments should further include the energy and materials required for fiber reconstruction, filament or ink preparation, forming, and post-treatment, together with the functional performance delivered by the regenerated product [140]. Recovered fractions that do not meet the requirements of polymer remanufacturing may be used as fibers, powders, or fillers in cementitious materials.

### 3.5. Reuse in Cementitious Composites

**Figure 8:**
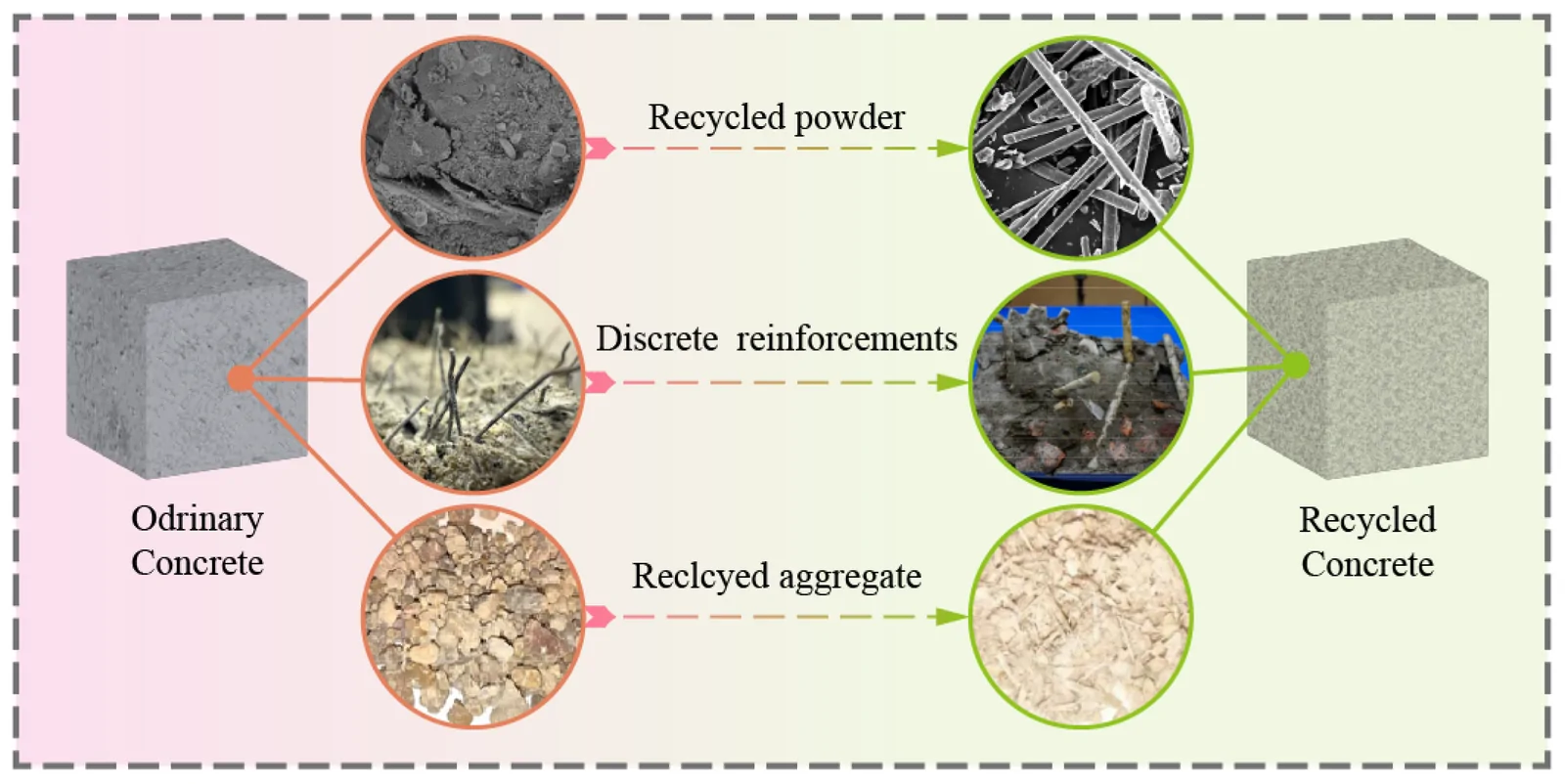
Waste FRP products in cementitious composites (figure elements sources: [200,201,202]).

Given that commercial FRP recycling is still dominated by mechanical shredding and crushing, the recovered products are typically obtained as fragments, shards, short fibres, or powders. Compared with rCFs, which require complex purification but retain higher value, rGFs generally exhibit lower economic attractiveness within the GFRP recycling chain, and their value is often insufficient to offset the costs associated with recycling and reprocessing [61]. In addition, the rapid development of the wind energy sector has generated substantial demand for the treatment of EoL-WTBs, which has also become a major source of GFRP waste. Compared with polymer matrices, cementitious composites can still be normally cast after incorporating untreated FRP fragments [72]. This indicates that cementitious composites exhibit good compatibility with recycled FRP products. As shown in Figure 8, through pathways such as aggregate substitution, discrete reinforcements, and the incorporation of functional fillers, recycled FRP can not only be efficiently valorized but can also reduce the production cost and carbon emissions of cement-based materials, thereby offering both environmental and economic benefits.

#### 3.5.1. Recycled Powder

**Figure 9:**
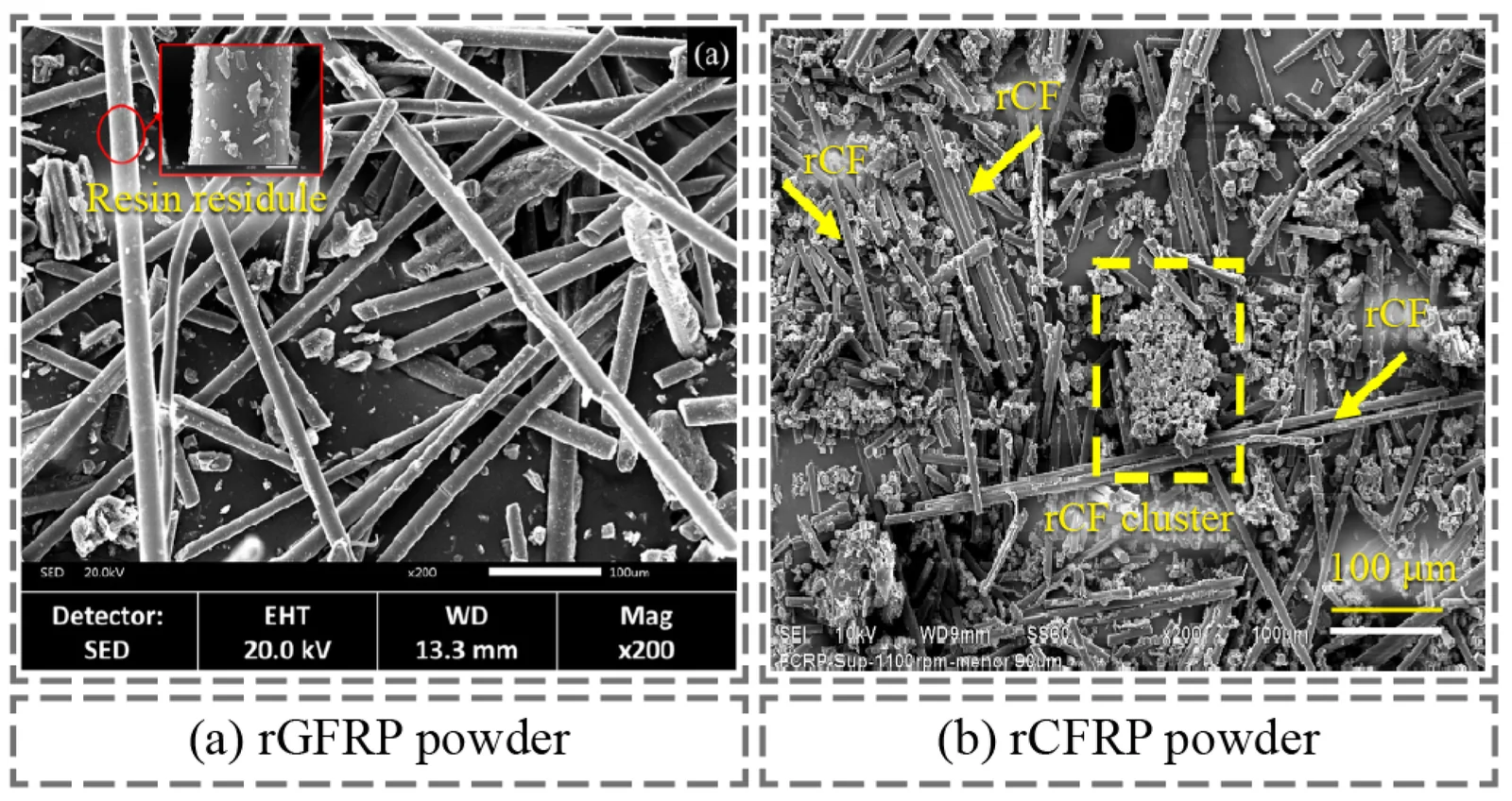
SEM morphologies of (**a**) rGFRP [200] and (**b**) rCFRP [212] powder.

As shown in Figure 9, recycled FRP that has been ground retains significant needle-like features, which enable it to function as a crack-bridging agent in cementitious composites [103,203], improving the drying shrinkage performance and pore structure of cementitious composites [204,205,206,207]. Additionally, the incorporation of recycled FRP powder can reduce the dry density of cementitious composites [97], offering a route for the development of lightweight recycled cementitious composites. However, the relatively poor interface wettability and irregular particle morphology of recycled FRP limit its performance. The incorporation of rFRP significantly increases the friction resistance within the system, hindering the orderly arrangement of particles and adversely affecting the flowability of the fresh mixture [208]. However, studies show that when the dosage is controlled to below 10% and the particle size is less than 600 µm, finely ground reclcyed FRP powder with good gradation promotes particle rolling and rearrangement within the composites, improving the flowability of the mortar to a certain extent [97,203,205,209]. When recycled GFRP powder is used as fine aggregate or as a cement replacement, it typically leads to a decline in the mechanical properties of cementitious composites; at low replacement ratios, compressive and flexural strength usually decrease by no more than 15% [48]. However, when used as a cement replacement at higher substitution rates, the decline in both compressive and flexural strength exceeds 30% [204]. Furthermore, due to the lower density of GFRP powder compared to mortar, it tends to float during the vibration process, affecting the uniformity of the formed material [103]. Despite these performance limitations, such recycled powders can still be used in non-load-bearing filling components or alkali-activated binder composites [210]. In engineering applications, by using FRP tubes as external confinement, the confinement effect can offset the negative impact of GFRP powder on the compressive capacity and freeze–thaw resistance of the core concrete, thus providing a technical pathway for its industrial application [211].

Unlike direct incineration, co-processing in cement kilns allows the waste GFRP powder to simultaneously serve as an alternative fuel and a partial raw material, achieving approximately 67% material recovery and 33% energy recovery [213], while reducing the residual waste that needs to be landfilled [214]. Additionally, the glass dust in GFRP contains some pozzolanic activity [215,216], which facilitates its resource utilization in cement composites. This technology requires pre-crushing the waste to an appropriate fineness [64] and systematic optimization to control pollution risks [4]. However, compared to direct incineration, co-processing in cement kilns can achieve a certain level of greenhouse gas reduction through the use of alternative fuels [65,136] and enable secondary recycling of the residual dust produced during incineration [150]. Cement kiln co-processing partially recovers both heat and fuel from the incineration process [217]. This technology has already been successfully applied commercially (e.g., Zajons Logistik, in Germany), with a rated disposal capacity of 60,000 tons per year [47]. However, this process is inherently a downcycling method; its core value lies in energy recovery, as GFRP is fully decomposed at high temperatures, and the residuals can only serve as low-value fillers, failing to retain the inherent high mechanical properties of the fiber-reinforced composites.

Additionally, when GFRP powder is used as an expansive agent, its volume expansion effect primarily arises from the chemical reaction between the residual polymer and the strongly alkaline pore solution in the cement paste. Due to factors such as GF surface resin coating, larger particle size of the powder, slow depolymerization of amorphous silica, and the tendency to form air pockets in alkaline environments, the gases generated during the early stages of the reaction directly cause volume expansion in the fresh mortar [205,218]. At the same time, benefiting from the water absorption capacity of GFRP powder, the bridging effect of residual rGFs, and the self-curing properties, the mechanical performance degradation induced by this process is comparable to that of commercial expansive agents [14]. Although the specific mechanisms are not yet fully understood, this approach still presents a potential pathway for functional value-added utilization of waste GFRP powder.

#### 3.5.2. Recycled Aggregate

Processing waste FRP into recycled aggregates for the partial replacement of natural coarse aggregates represents a feasible pathway with both strong potential for large-scale waste utilization and significant environmental benefits [30,219]. This approach can effectively retain the inherent high-strength characteristics of FRP, with compressive strength reaching as much as 187.08% of that of natural aggregates [220]. However, the primary challenge of this technique lies in the fact that the incorporation of GFRP- or WTB-derived recycled aggregates can significantly deteriorate the ITZ of concrete, thereby reducing material stiffness [30]. Studies have shown that, at a replacement ratio of 6–10%, the elastic modulus of concrete decreases by approximately 20% [33]. The underlying reason is that polymer-based particles possess relatively low elastic modulus and compressive strength, making them more prone to deformation under compressive loading, which in turn weakens the overall efficiency of stress transfer and load-bearing capacity [221]. This ITZ deterioration effect also renders current design codes unable to accurately predict the mechanical properties of such materials, particularly compressive strength and elastic modulus [33,222]. Therefore, it is of critical importance to establish modified mechanical models that explicitly account for ITZ deterioration effects.

**Figure 10:**
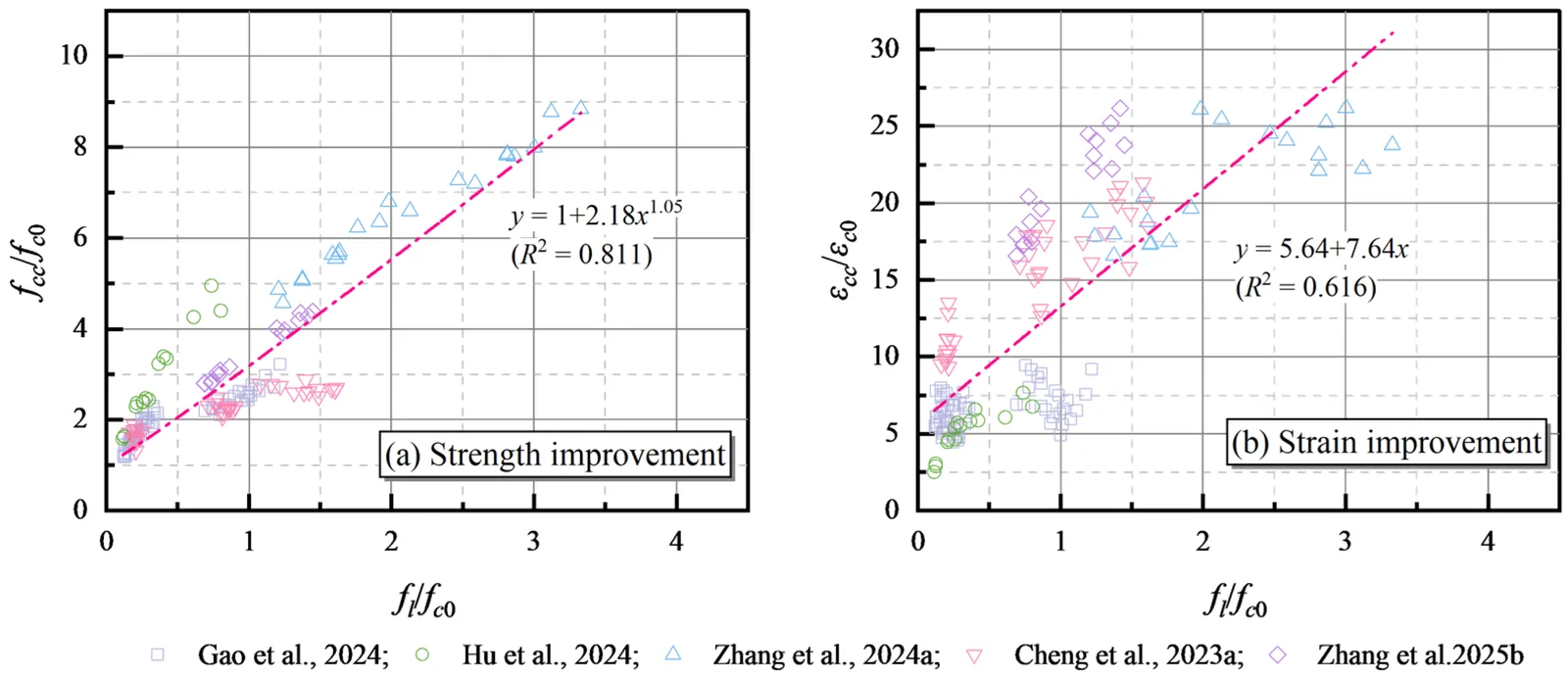
(**a**) Strength and (**b**) strain improvement of confined cylinders with rFRP aggregates [220,225,226,227,228].

To compensate for the aforementioned performance deficiencies, the use of lateral confinement has proven to be an effective means of enhancing load-bearing capacity. The resulting strength enhancement is primarily attributable to the external confinement effect, rather than to any intrinsic improvement in the core concrete itself [223,224]. For example, the compressive strength of concrete with 100% recycled GFRP aggregate replacement can increase by 67–198% with increasing confinement ratio defined in Equation (1) [28,225]. As shown in Figure 10, the strength improvement of concrete cylinders follows a pronounced exponential relationship with an *R*^2^ of 0.811 [220,225,226,227,228], which is generally consistent with that of conventional confined concrete [229]. This indicates that the existing strength design criteria for confined concrete remain applicable to such materials. However, the incorporation of recycled FRP aggregates renders the deformation behavior of concrete more complex, and the evolution law of its ultimate strain remains insufficiently understood; accordingly, the corresponding deformation-based design criteria still require further refinement.

(1) $$\frac{f_{l}}{f_{c0}}=\frac{2E_{f}t_{f}\varepsilon_{h,rup}}{Df_{c0}}$$

where *f_l_* and *f_c_*_0_ are the strengths of lateral confinements and core concrete, respectively, and both of their units are MPa. *E_f_*, *t_f_*, and *ε_h,rup_* are the elastic modulus (MPa), thickness (mm), and rupture hoop strain of the lateral FRP confinement, respectively. *D* is the diameter of the whole FRP-confined cylinder (mm).

To further enhance sustainability and address the contradiction that conventional fully wrapped FRP jackets generate secondary waste after service, increasing attention has been paid to the innovative concept of directly using waste FRP components, such as EoL-WTBs, as an integral confinement system. In this approach, the FRP shell provides effective lateral confinement to the concrete, with a mechanism similar to that of newly manufactured FRP tube-concrete members [230]. Without relying on a high-strength ITZ, the structural performance can be improved through the combined action of normal pressure and friction. This strategy not only avoids the use of virgin FRP materials but also simultaneously reduces economic cost and environmental burden through the direct utilization of waste components. Nevertheless, lateral confinement technology still faces several limitations in engineering practice, including restricted applicability (e.g., insufficient investigation of non-circular members), unclear long-term performance, and the potential generation of new waste over the full life cycle. Therefore, future development should move beyond a single structural strengthening approach and instead promote multiscale collaborative innovation in material design, interfacial regulation, and structural configuration, to establish integrated technical pathways that are mechanically reliable, environmentally compatible, and sustainable over the full life cycle.

#### 3.5.3. Discrete Reinforcements

In addition to replacing natural aggregates, shredded or crushed FRP can also be used as discrete reinforcements, incorporated into cementitious composites in the form of recycled micro-/macro-fibers, to enhance their mechanical properties, ductility, and internal structure [29,231,232,233,234,235,236,237,238,239]. Studies have shown that incorporating 2 wt.% rCFs can increase the 28 d compressive strength of concrete by 14.8%, while the elastic modulus only slightly decreases by 5.8%, demonstrating excellent crack-bridging behaviors [234]. Shredded or crushed fragments of GFRP, obtained from sources like EoL-WTBs, can also effectively enhance the properties of concrete, with their tensile-related performance being controlled by both the strength of the matrix itself and the bond strength between the GFRP fragments and the matrix [29,240]. These findings suggest that, through proper morphological design and interfacial control, recycled FRP fibers can play a clear reinforcement role in cementitious composites and offer potential for value-added applications [241,242]. Similarly, a rough surface of rCFs caused by thermal or chemical recycling, along with the increase in oxygen-containing functional groups, enhances their interfacial bonding with the cement matrix, giving the composite advantages in tensile-related properties and dispersibility [106,243,244,245]. Furthermore, at high temperatures, the cement matrix, as an external coating layer, prevents the oxidation of rCFs [58], allowing the rCFs reinforced concrete to retain 49.19% of its flexural strength after high-temperature exposure [104]. Meanwhile, rCFs can enhance the durability and electrical properties of cement-based systems through effective crack-bridging and interfacial interactions. Compared to using vCFs, in rCF-based self-sensing composites, the global warming potential (GWP) and cost can be reduced by 13.69–75.36% and 36.5–70.0%, respectively [55,245]. Based on these characteristics, rCFs have good application prospects in structural health monitoring, multifunctional smart concrete, and transportation infrastructure, among other fields [207,246], facilitating the value-added utilization of waste CFRP and reducing EoL disposal burdens.

**Figure 11:**
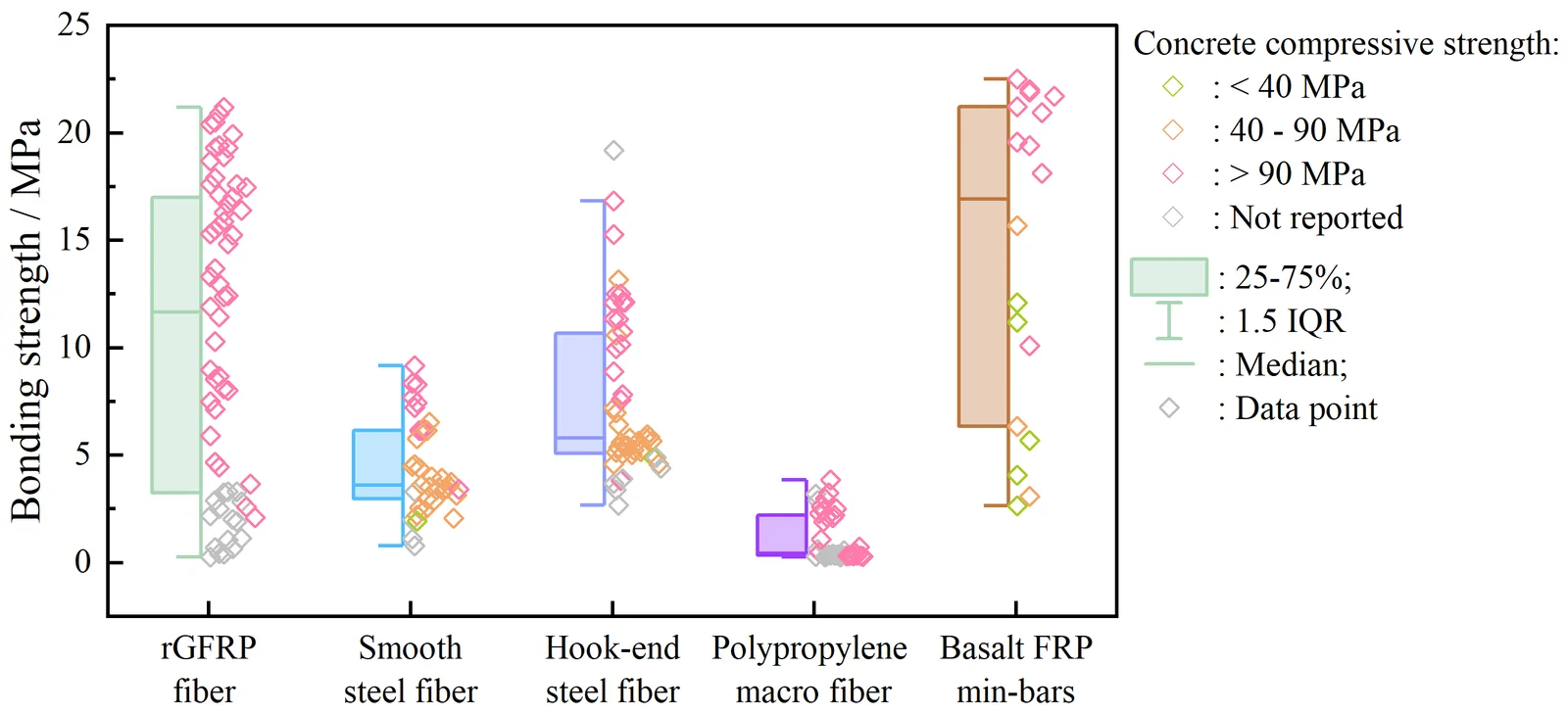
Bond strength of recycled GFRP fibers and engineering macro fibers [247,248,249,250,251,252,253,254,255,256,257,258,259,260,261,262].

To further evaluate the engineering potential of mechanically recycled FRP as reinforcement for cementitious composites, Figure 11 compares the reported interfacial bond strengths of rGFRP with those of established engineering macro fibers [247,248,249,250,251,252,253,254,255,256,257,258,259,260,261,262]. The reported bond strength of rGFRP ranges from approximately 0.28 to 21.2 MPa, with a median of 11.66 MPa, covering a broader interval than most of the engineering fibers considered. Such variability is associated with the different preparation and interfacial modification strategies adopted in previous studies, including mechanical crushing, controlled cutting, and surface treatment [248,252,255,263]. The lower range of rGFRP overlaps with polypropylene fibers and smooth steel fibers, whereas the upper-end values extend into the principal ranges attained by hooked-end steel fibers and BFRP mini-bars. More importantly, the high bond strengths achieved by appropriately processed rGFRP indicate that its interfacial performance can be effectively regulated through suitable processing and interface treatment strategies [252,263]. Therefore, rGFRP can develop an interfacial stress transfer capacity comparable to that of established engineering macro fibers, supporting its potential use as discrete reinforcement in cementitious composites.

Recycled fibers often have residual matrix material attached to their surfaces, which is difficult to completely remove. The roughened surface of the fibers can enhance their interlocking with cementitious matrices [29,55], whereas the heterogeneous distribution of residual matrix and surface features can result in non-uniform fiber–matrix ITZ properties, leading to significant variability in the quality of recycled products [169]. Moreover, due to differences in the source, surface morphology, impurities, and recycling processes, the surface characteristics of recycled fibers exhibit significant variation, making their dispersion behavior more complex than that of virgin fibers [63,264]. Additionally, the large specific surface area of fibrous recyclates obtained from FRP crushing can increase free-water adsorption, thereby adversely affecting the rheology and workability of fresh mixtures [73,265]. For rCFs, surface treatments can improve wettability through the removal of residual resin and the introduction of oxygen-containing or other hydrophilic functional groups. In addition, dispersant-assisted modification can suppress fiber agglomeration through electrostatic repulsion and steric hindrance [266,267,268]. For rGFRP from crushing and shredding wastes, the accompanying fine resin powder, despite having a lower specific surface area, exhibits strong hygroscopicity, further exacerbating the deterioration of workability. Currently, feasible technical approaches to address these issues include effectively separating rGFRP fibers from waste powders or modifying the surface of fibers using silane coupling agents. By forming hydrophilic groups on the surface of recycled fibers, the hydrophilicity of the fibers can be enhanced, thereby improving the attachment efficiency of hydration products on the fiber surface and enhancing both the bonding performance between fibers and the matrix, and the dispersion performance of recycled fibers themselves [45,46,263].

Compared to recycled microfibers, rFRP needles, which are prepared from cutting waste FRP members, exhibit a more significant crack-bridging effect and share the responsibility of bearing external loads with the component. These needles do not require the removal of surface residual resin during application, and their effect can be further enhanced with an increase in fiber volume fraction and anchoring length [31,269,270]. Additionally, since rFRP needles are not hydrophilic, the deterioration of the slump of concrete does not become significant with increasing fiber content [32]. Incorporating 1.5 vol.% of rGFRP needles can increase the concrete tensile strength by 52% [271], significantly improving material toughness and energy absorption capacity. However, this may also result in a slight decrease in compressive strength due to weak interfacial bonding [272]. Moreover, the surface morphology of rFRP needles plays a crucial role in contributing to their mechanical properties. rGFRP needles with a helical surface texture can enhance the shear strength of concrete beams by 8–10% through mechanical interlocking, while smooth-surfaced rGFRP needles may experience performance degradation due to insufficient interlocking, thus failing to fully realize their inherent strength [31].

**Figure 12:**
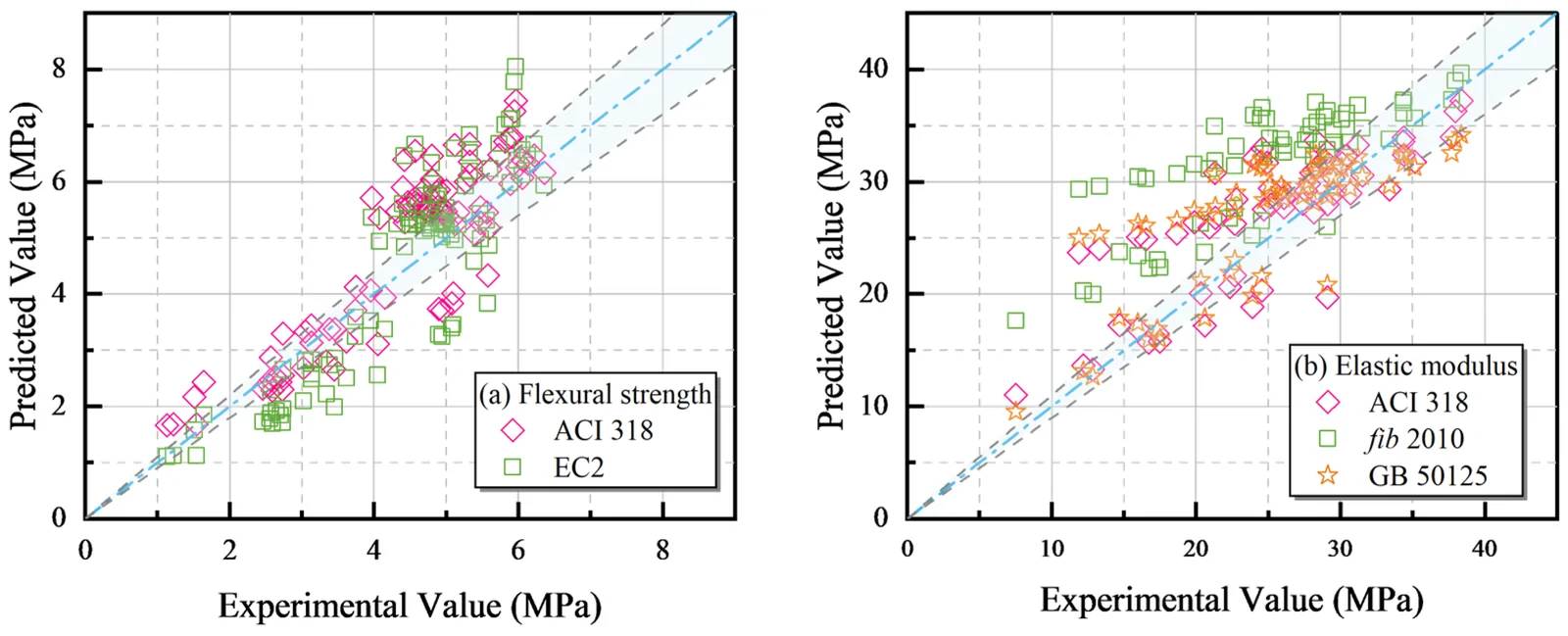
Prediction results of (**a**) flexural strength and (**b**) elastic modulus via current codes. (Data collected from [8,29,31,32,203,220,225,226,227,228,235,240,269,277,278].)

Although recycled FRP (rFRP) can effectively enhance the tensile, bending, and post-crack properties of cement-based materials as discrete reinforcements, its engineering application is still limited by the incompatibility with current design codes. Analysis results listed in Table 3 reveal that existing codes [273,274,275,276] generally suffer from low determination coefficients and large prediction errors when forecasting the bending strength and elastic modulus of materials incorporating such reinforcements. Notably, as shown in Figure 12, the predictions for elastic modulus tend to be overly optimistic, as the current codes fail to account for the degradation in deformation properties of cementitious composites due to the weak ITZ of recycled fibers and GFRP needles. As a result, mechanical models established based on traditional homogeneous materials are still inadequate in accurately characterizing and quantifying the actual mechanical contributions of recycled reinforcements, thereby limiting their reliable design and standardized application in practical engineering.

#### 3.5.4. Challenges in Reuse in Cementitious Composites

Recycled FRP products have been used in conventional mortar and concrete, ultra-high-performance concrete, and other cementitious composites [55,63,74,232,279,280]. Their functions depend on the recovered form. rGFRP fragments can act as low-cost discrete reinforcements for crack bridging and toughening. rGFRP powder can be used as a precursor in alkali-activated cementitious composites. Conductive rCFRP can be incorporated into self-sensing cementitious composites and has shown satisfactory dispersion in cementitious matrices. However, while recycled GFRP fibers and fragments can improve tensile and post-cracking performance, their bond and pull-out behavior in cementitious matrices remains unclear [263,281]. Variations in residual resin, wettability, morphology, and anchorage length affect adhesion, friction, and mechanical interlocking, limiting the development of reliable bond-slip models and design methods.

The use of recycled FRP in cementitious composites can reduce virgin-material consumption and retain part of the functional value of the waste. Recycled GFRP used as fine or coarse aggregate can replace natural resources such as river sand. Its incorporation may also avoid emissions associated with landfilling or incineration and reduce the demand for virgin materials [204,282,283]. The life-cycle assessment results listed in Table 4 indicate potential reductions in carbon emissions and engineering costs. Quantitative evidence remains limited for full life-cycle emissions, processing energy consumption, industrial scalability, supply stability, and engineering cost boundaries [74].

These environmental benefits depend on whether the resulting composites satisfy basic engineering requirements. The influence of recycled FRP varies with its form, dosage, and compatibility with the required mechanical performance. Powder and aggregate can accommodate relatively large quantities of waste, although their mechanical contribution is limited and high replacement levels may reduce workability, stiffness, or strength. Fibers and fragments can improve crack bridging and post-cracking load-bearing at lower dosages. Their use may provide a more favorable balance between waste utilization and mechanical performance.

## 4. Current Challenges

**Figure 13:**
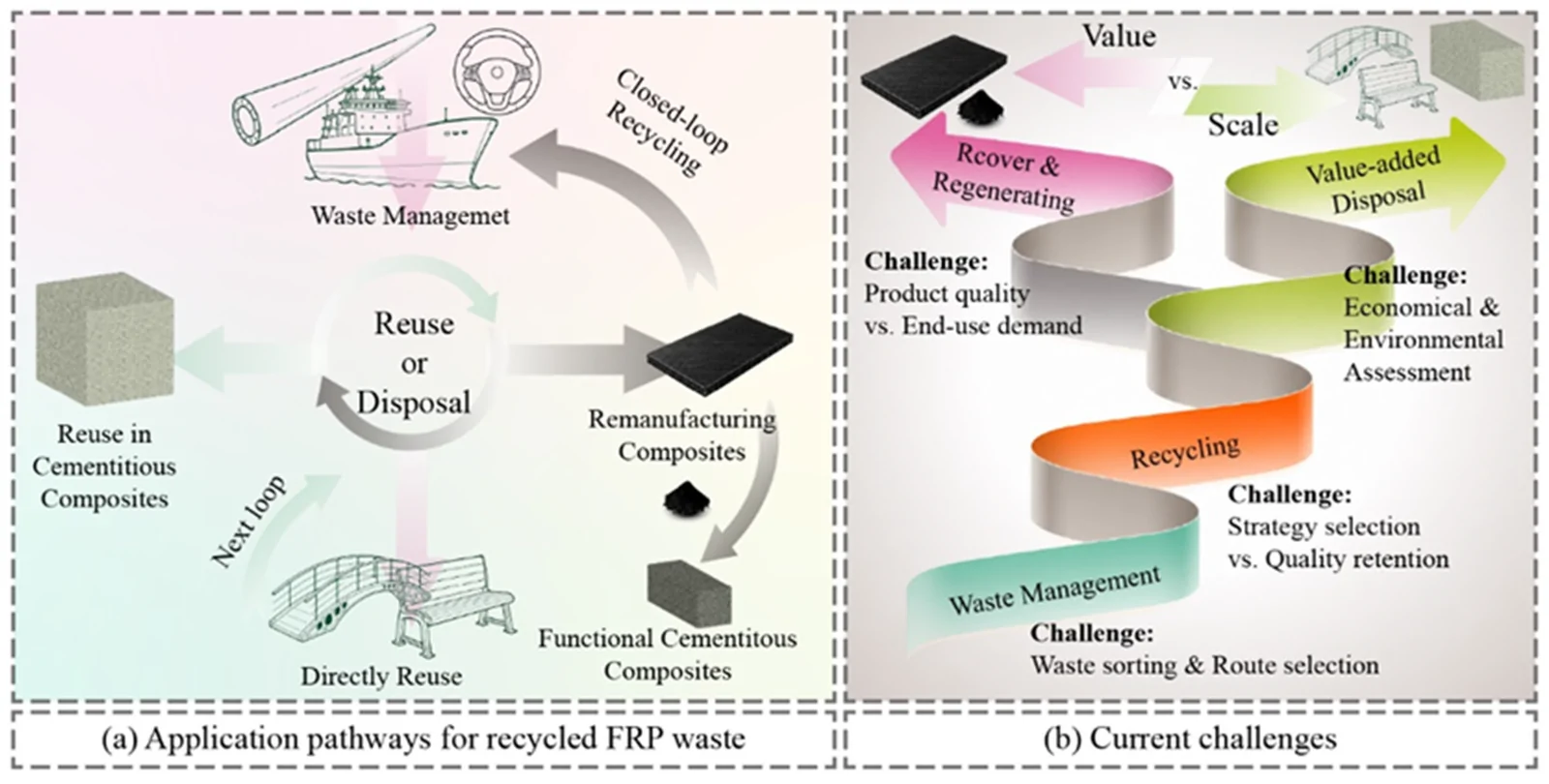
(**a**) Application pathways and (**b**) key challenges for recycled FRP waste.

Current FRP recycling and disposal research has developed several significant routes. As shown in Figure 13, these routes should be understood as a decision process governed by the balance between value retention and large-scale utilization. The main unresolved challenges identified in this review are summarized as follows.

- Recycling route selection should be linked to end-use requirements through graded allocation of recovered FRP. Recycling routes are commonly evaluated in terms of energy consumption, cost, fibre-property retention, processing efficiency, and technology readiness. Their practical value, however, also depends on whether the recovered material is suitable for its intended application. Decommissioned components with adequate residual capacity should be directly reused; high-value fibers with limited damage, sufficient length, stable mechanical properties, and suitable surface conditions should be directed toward polymer-composite remanufacturing; and heavily damaged, shortened, contaminated, or lower-value GFRP fractions should be used in cementitious composites as aggregates, fillers, or discrete reinforcements. This graded allocation strategy matches recovered product quality and intrinsic value with downstream requirements while retaining as much of the original functional value as possible.
- A graded matching relationship between recovered FRP products, regeneration routes, and end-use requirements has not yet been established. The reutilization potential of recycled FRP depends on the combined attributes of the recovered product, including fiber type and unit value, fiber length distribution, mechanical properties, surface condition, residual matrix content, contamination, product form, processability, and batch consistency. FRP remanufacturing offers greater potential to retain the functional and economic value of recovered fibers, but it requires tighter control of recovered-material quality and matrix compatibility. Cementitious composites may accommodate lower-grade fractions containing short-cut or milled recycled fibers, irregular fragments, or residual resin, although their suitability must still be verified against performance and durability requirements. A graded utilization framework linking recovered product quality, matrix selection, processing route, and application requirements is therefore needed.
- Environmental and economic assessments remain difficult to compare. Existing studies use different system boundaries, functional units, energy sources, processing capacities, and substitution assumptions. Some assessments consider only fiber recovery, whereas others include pretreatment, remanufacturing, transportation, or post-treatment. Consequently, the reported environmental and economic benefits cannot always be compared directly. Future assessments should include the main recycling and regeneration stages and relate environmental impacts to the functional performance of the regenerated product.

## 5. Conclusions

Research on FRP recycling and remanufacturing has clarified many of the technical, environmental, and economic trade-offs associated with different recycling strategies and end-use utilization. The main conclusions of this review are as follows:

(1) Mechanical, chemical, and thermal recycling each have limitations in terms of recovered material quality, operating conditions, post-treatment requirements, cost, and environmental impact. However, innovative ITZ separation methods, including freeze–thaw cycling, pulsed-laser treatment, and hygrothermal coupling, show potential for broader applicability. Although research remains limited, these methods represent a promising pathway for overcoming the limitations of conventional recycling technologies.
(2) Direct reuse best preserves the structural value of decommissioned FRP components by avoiding separation and retaining their capacity and strength. However, its applicability is constrained by component geometry, residual condition, transport and installation requirements, and the lack of standardized design methods. It should therefore be prioritized only when these conditions are satisfied.
(3) The value retained through FRP remanufacturing is controlled by both recovered fiber quality and subsequent processing conditions. Recycled fibers can be reorganized into nonwoven mats and additive manufacturing for regenerated composites. Fiber length, surface condition, residual matrix, and interlaminar defects jointly determine the final performance. Current studies mainly focus on forming parameters and post-treatment, whereas transferable relationships among recovered-fibre quality, interfacial behaviour, processing conditions, and composite performance remain insufficiently established.
(4) Cementitious composites provide a comparatively tolerant and scalable route for utilizing heterogeneous recycled FRP products. Aggregate and fragment substitution can accommodate relatively large waste volumes; recycled powders generally retain less of the original fibre value, but may provide filling, thermal, electrical, or other functional effects; short fibres and FRP fragments can serve as discrete reinforcements to bridge cracks and therefore improve post-cracking resistance. However, the manufacturing and structural utilization still require further clarification of bond behaviour, durability, and appropriate design parameters.
(5) Recovered FRP should be allocated according to its intrinsic value and degree of damage. Decommissioned components with adequate residual capacity should be directly reused; high-value fibers with limited damage and stable properties should be directed toward FRP remanufacturing; and heavily damaged, shortened, contaminated, or lower-value GFRP fractions should be utilized in cementitious composites as aggregates, fillers, or discrete reinforcements. This graded allocation strategy can retain the functional value of FRP waste while matching recovered-material quality with appropriate end-use requirements.
(6) Future research should prioritize application-oriented matching among recycling quality, remanufacturing routes, and end-use functions. The principal challenge in the current FRP recycling and utilization lies in the lack of a comprehensive, graded utilization framework that connects recycling quality, molding systems, and end-use applications. The current bottleneck is not only in the performance fluctuations of recycled materials and remanufacturing process defects, but also in the lack of synchronised development of performance prediction models, design standards, regulatory certifications, raw material supply, and market access systems.

## Figures and Tables

**Table 1:** Reported industrial and pilot-scale implementations of FRP recycling technologies and their available operational characteristics.

Agency	Region/Country	Strategy	Reported Industrial Information	Ref.
Anshu Langqing Environmental Protection Equipment Co., Ltd.	China	Machine shredding	Product: GFRP shards with lengths of 10–30 mm and widths of 0.6–4.75 mm.	[45]
Henan Ruinuo Environmental Protection Equipment Co., Ltd.	China		Product: GFRP shards with particle sizes of 0.3–2.36 mm, available for specimen preparation.	[46]
Procotex	Belgium		Product: milled rCFs up to 120 mm in length and recycled aramid fibers with lengths of 0.25–120 mm. Status: products commercially available since 2014.	[47]
Retsch GmbH	Germany		Product: GFRP powder with an average particle size of 0.39 mm and a fineness modulus of 1.64.	[48]
Rivierasca S.p.A.	Italy		Product: recycled GFRP fibers up to 50 mm in length and approximately 13 μm in diameter.	[49]
Recycle Co., Ltd.	China		Product: recycled WTB-GFRP obtained after separation of balsa wood and polymer powder.	[50]
Tianjin Longjin Energy Saving Technology Co., Ltd.	China		Product: GFRP powder processed to 200 mesh.	[51]
Wittmann Technology GmbH	Austria		Capacity: 100–500 t/year.	[52]
Adherent Technologies Inc.	U.S.	Thermal	Process: catalyst-assisted vacuum pyrolysis achieved approximately 99% recovery of rCFs.	[53]
CFK Valley Stade Recycling GmBH & carboNXT GmBH Stade	Germany		Capacity: more than 1000 t/year, since operation launched in 2011. Product: milled rCF of 80–500 μm and pelletised/chopped products of 1–100 mm.	[47]
Carbon Fiber Recycling LLC	U.S.		Product: rCF with a density of 1.81 g/cm^3^, a length of 1.5 ± 1.2 mm, a diameter of 6.7 ± 0.8 μm, and a sizing content of 0.99%.	[54]
ELG Carbon Fibre	U.K.		Product: typical rCF diameter of ~7 μm, length of ~6 mm, tensile strength of 4150 MPa, and tensile modulus of 230–255 GPa. Capacity: 2000 t/year recovered rCF output. Status: commercial CFRP recycling by pyrolysis.	[47,55]
Japan Chemical Fibers Association (JCFA)	Japan		Capacity: 700 t/year.	[36]
Nantong Fuyuan Carbon Fiber Recycling Co., Ltd.	China		Product: 81.85% of the recovered rCFs had lengths of 2–5 mm.	[56,57]
Tencate Advanced Composites	The Netherland		Product: recovered fibers with lengths of 5–400 μm and a diameter of ~7 μm. Performance: ~90% mechanical property retention.	[58]
Innoveox	France	Chemical	Process: near-/supercritical fluid solvolysis of CFRP. Status: led the RECCO project for pilot-scale process development following laboratory-scale optimization.	[59]
Panasonic Electric Works Co., Ltd.	Japan		Process: subcritical-water hydrolysis of FRP. Product: recovered resin components reused for recycled resin and low-profile additives. Capacity: pilot plant, 400 kg/operation.	[59,60]
Zajons Logistik	Germany	Special	Process: compocycle co-processing of shredded GRP with RDF in cement kilns. Product: mineral fraction serves as cement feedstock, while the polymer fraction provides calorific value. Capacity: 60,000 t/year.	[47]

Note: Reported information includes processing capacity, recovered-product characteristics, recovery performance, and implementation status. Data for equipment suppliers refer to FRP materials processed using their equipment.

**Table 3:** Empirical models and calculation results from current codes. (Data collected from [8,29,31,32,203,220,225,226,227,228,235,240,269,277,278].)

Item	Code	Formulas	*R* ^2^	RMSE	MAPE/%
Flexural strength	ACI 318-19 [274]	$0.94f_{c}^{0.5}$	0.826	0.78 MPa	14.11
	EC2 [275]	$\max\left\{\left(1.6-h/1000\right),1\right\}\times 0.33f_{c}^{0.67}$	0.816	0.91 MPa	22.38
Elastic modulus	ACI 318-19 [274]	$4700\sqrt{f_{c}}$	0.644	4.59 GPa	17.29
	*fib* 2010 [273]	$21500{\left(f_{c}/10\right)}^{1/3}$	0.636	7.93 GPa	34.48
	GB 50125-2012 [276]	$\frac{10^{5}}{2.2+\frac{34.7}{f_{c}/\alpha_{ct}}}$	0.601	4.82 GPa	17.73

Note: *h* is the depth of specimens (mm), and *α_c_*_1_ is the coefficient for determining the axial compressive concrete strength (*f_c_*), which depends on the strength grade of concrete. *RMSE* and *MAPE* are root mean squared error and mean absolute percentage error.

**Table 4:** Summary of reported environmental, economic, and engineering outcomes of recycled FRP in cementitious composites in the past 5 years.

Ref.	Time	FRP Type	Application	Reported Outcomes
[284]	2025	GFRP	Powder (raw)	Cost: decreased by ~21% for low-income concrete relative to the study reference.
[285]	2025	WTB-GFRP	Fibers and aggregates	GWP: decreased by 11.54%. Cost: decreased by up to 9%. Strength: flexural strength increased by 15%.
[18]	2025	WTB-GFRP	Fibers	GWP: decreased by 28.3%; abiotic depletion potential for fossil fuels decreased by 5.9%; overall environmental impact decreased by 0.2%, for 1 m^3^ concrete.
[65]	2025	WTB-GFRP	Rebars	GWP: 2.4 kg CO_2_-eq/kg GFRP waste of emissions avoided at a recycling capacity of 100 kg/h. Cost: decreased by 92.5% relative to steel rebars.
[18]	2025	WTB-GFRP	Aggregates	GWP: decreased by 3.21% at 10% GFRP aggregate content relative to the reference mixture.
[55]	2024	CFRP	Fibers	GWP: decreased by 75.36%. Cost: decreased by 36.5% for alkaline-activated self-sensing cementitious composites relative to the corresponding reference.
[8]	2024	WTB-GFRP	Fibers	GWP: decreased by 0.12 kg CO_2_-eq/(MPa·m^3^) at 1.5% rGF (compressive-strength basis); −7.5% at 6.0% rGF (flexural-strength basis).
[286]	2023	WTB-GFRP	Aggregate	GWP: increased by 194 kg CO_2_-eq for aggregate replacement due to cutting and grinding, while −4516 kg CO_2_-eq per reported functional unit for cement replacement.
[282]	2022	GFRP and CFRP	Fibers	GWP: reduced 18–25% green-house gas Cost: increased 20% relative cost to conventional concrete.
[270]	2022	GFRP and CFRP	Fibers	GWP: reduced up to 13.8% CO_2_ emissions, accompanied by increased compressive and flexural strengths under the reported optimum condition.
[239]	2021	rCFs	Fibers	GWP: reduced CO_2_ emissions by 22%. Strength: compressive strength, flexural strength, and impact resistance increased by 5.5%, 38.8%, and 406%, respectively, with 1.5 vol.% rCF dosage.
[245]	2020	rCFs	Fibers	GWP: decreased by 13.69%. Cost: reduced by 70% when 1% rCFs were used instead of vCFs in cementitious composites.

Note: Reported values retain the original functional units and comparison bases and should not be directly compared across studies.

## Data Availability

No new data were created or analyzed in this study. Data sharing is not applicable to this article.

## Appendix A. Morphological and Mechanical Quality of Recovered Fibers

**Table A1:** Morphological and mechanical quality of recovered fibers [5,66,76,77,81,82,85,86,87,89,90,91,99,100,104,105,108,109,113,114,115,116,118,124,127,129,287].

Ref.	Matrix/Fiber	Process	Surface and Structure Quality	Fiber Mechanical Retention
Ahmed et al. (2025) [127]	EP/GF	Water-based freeze–thaw cycling for 10 d; each cycle comprised 30 min at 25 °C, 8 h at 50 °C and 16 h at −30 °C	Fiber architecture was retained without severe visible surface damage; crack-volume fraction increased by 65.3% and connected porosity increased by 32.3%, indicating selective interfacial separation.	Nanoindentation hardness 92.66%; Elastic modulus: 95.99%
Chen et al. (2025) [77]	EP/E-GF	Supercritical ethanol at 275–325 °C for 40 min with 1.0–1.5 wt.% NaOH	Smooth and intact at 275 °C; elevated temperature caused rGF surface damage	Strength: 30.40–95.64%; Elastic modulus: 94.82–112.50%; Rupture strain: 27.42–76.76%
Hao et al. (2025) [87]	EP/GF	Aqueous Zn(OAc)_2_ treatment at 250 °C for 5 h; 20 wt.% catalyst; GFRP/solution ratio of 1:5	After removal of the degraded matrix polymer, rGF exhibited a clean, smooth surface and approximately the same diameter as the original GF.	N. A.
Liu et al. (2024) [85]	EP/GF	Microwave swelling followed by FeCl_2_/TBHP oxidation at 60 °C	Clean and smooth surface comparable to virgin GF; no visible resin residue	Strength: 97.0%
Ahrens et al. (2023) [89]	EP/GF & EP/CF	6 wt.% triphos-Ru-TMM and 8 vol.% iPrOH in toluene at 160 °C for 3 d; 6 d for large treatment	Recovered GF remained smooth, and diameter decreased by 5.26%; the primer was partially removed, and no epoxy residue was detected. Recovered CF diameter was 6.5 ± 0.9 μm	Strength: 86.27%; Elastic modulus: 95.52%
Ragupathi et al. (2023) [124]	PP/E-GF	20 kHz ultrasonic pre-cracking, peel separation, and ultrasonic reconsolidation; optimum reconsolidation time 250 ms, amplitude 39 μm, and force 300–500 N	Fiber length, orientation, and layer dimensions retained; separated layers showed minor scratches, residual PP, and fiber-bundle waviness; 500 N reconsolidation eliminated visible interfacial gaps and pores	N. A.
Xu et al. (2023) [99]	EP/E-GF	Pyrolysis at 500 °C for 60 min under N_2_, 20% H_2_O/N_2_ or 20% CO_2_/N_2_; post-oxidation at 500 °C for 30 min	H_2_O promoted resin cracking and produced the cleanest surface; local char granules remained on the N_2_- and CO_2_-derived fibers	Strength: 94.03%/83.98% Elastic modulus: 100.57%/99.80% Rupture strain: 93.64%/83.64% (under H_2_O/CO_2_, compared with N_2_ atmosphere) recovered 94.03%, 83.98
Rani et al. (2022) [118]	EP/E-GF	Immersion in H_2_O_2_/acetic acid for 30 min; 700 W microwave treatment for 180 s; ultrasonic washing and drying	Predominantly clean and smooth surfaces; maximum EP decomposition was 97.2%	Strength: 97.63–99.77%; Elastic modulus: 91.43–93.33%; Rupture strain: 94.49–95.44%
Rahimizadeh et al. (2020) [66]	E-GF	Milling	Short bundles and filaments with abundant epoxy residue; heterogeneous surface; with an *Ra* of 63.1–88.6 nm	Strength: 44.6–80.9%; Elastic modulus: 96.1–107.0%; Rupture strain: 40.7–75.0%
Rahimizadeh et al. (2020) [66]	E-GF	Pyrolysis at 550 °C for 45 min under N_2_; oxidation at 550 °C for 10 min	Sizing degraded; rug-like surface with local resin residue; with an *Ra* of 12.66–18.73 nm	Strength: 34.6–39.3%; Elastic modulus: 118.2–120.3%; Rupture strain: 28.5–35.1%
Pender & Yang (2017) [114]	EP/E-GF	Air treatment without catalyst at 500 °C for 80 min; CuO at 430 °C for 65 min; CeO_2_ at 450 °C for 60 min; Co_3_O_4_ at 450 °C for 65 min	Fibers were liberated at lower furnace temperatures with metal oxides; polymeric sizing was degraded. CuO-assisted degradation produced a transient surface-temperature peak of approximately 620 °C.	Tensile strength retained 36.86% when without catalyst; 43.53%, 44.71%, and 38.82, when using CuO, CeO_2_, and Co_3_O_4_, respectively
Chen et al. (2025) [77]	EP/CF	Supercritical ethanol at 275–325 °C for 40 min with 1.0–1.5 wt.% NaOH	Roughened and grooved surface with Na and N element residues	Strength: 91.58–99.24%; Elastic modulus: 94.60–108.35%; Rupture strain: 90.91–103.03%
Cheng et al. (2025) [113]	EP/CF	Cr_2_O_3_-coated honeycomb at 520 °C for 45 min under 80% O_2_; 180 mL/min	About 98.01% EP decomposition; fluffy and disordered rCF; cut-fiber mean length of 2.8 mm; more than 80% of fibers between 2.0 and 3.5 mm; epoxy-compatible sizing partly retained	Interfacial shear strength maintained 93.8% with EP and 91.0% with PLA
Li et al. (2025) [129]	EP/CF	Two-step 1064 nm nanosecond laser treatment: delamination under N_2_ followed by oxidative cleaning in air; optimized defocus +130 mm, step-1/step-2 powers of 420/120 W; total time 147 s	Original woven architecture and dimensions retained; resin removal reached 97.92%; optimized energy compensation limited etching, microcracks, micropores, and local delamination.	Strength: 93.38%; Elastic modulus: 105.77%
Tortorici et al. (2025) [287]	EP/CF	Aqueous H_2_SO_4_ solvolysis at 75 °C for 17 h under atmospheric pressure	Matrix removal above 99%; no visible fiber damage; sizing removed; *Ra* increased 46.09% (from 9.46 to 13.82 nm)	N. A.
Zhao et al. (2025) [86]	EP/CF	NMP preswelling followed by FeCl_3_-catalyzed hydrolysis at 170 °C for 3 h	Matrix removal of 99.3%; longitudinal grooves preserved without erosion; *Ra* increased by 5.54% (from 289 to 305 nm)	Strength: 95.7%; Elongation: 97.3%
Dai et al. (2024) [115]	EP/CF	Ar pyrolysis at 400–800 °C for 10–40 min, followed by air oxidation at 450–600 °C for 10–40 min	Optimized conditions produced clean rCF surfaces; oxidation removed pyrolytic carbon, whereas the pyrolysis parameters mainly affected graphitization	Strength: 96.05%
Liu et al. (2024) [85]	EP/CF	Microwave swelling followed by FeCl_2_/TBHP oxidation at 60 °C	Clean and smooth surface; trace Fe detected; graphitic structure remained close to virgin CF	Strength: 95.6%
Okada et al. (2024) [5]	EP/CF	Electrolytic sulfuric acid treatment at 100–150 °C	Continuous form retained without reported cracking; acidic surface groups increased; fibers longer than 70 m demonstrated at tank scale	Strength: ~100%
Vogiantzi & Tserpes (2024) [82]	EP/CF	Water solvolysis at 320–400 °C and 115–300 bar for 5–90 min	Continuous and generally clean fibers without visible cracks, with a diameter loss of 1.71–11.57%	Strength: 68.8–93.6%; Elastic modulus: 61.4–81.9%
Vogiantzi & Tserpes (2024) [82]	EP/CF	Acetone solvolysis at 300–350 °C and 85–230 bar for 40–60 min	Continuous fibers; visible resin residue remained at 300 °C and 85 bar	Strength: 75.2–80.5%; Elastic modulus: 66.2–69.5%
Xu et al. (2024) [100]	EP/CF	N_2_ pyrolysis at 400–600 °C for 10–60 min; post-oxidation at 500 °C for 40 min	Post-oxidation removed surface char and produced clean, smooth fibers; severe pyrolysis reduced the protective char layer and increased direct oxidative erosion.	Strength retention: 85.79–94.36%; optimum 94.36% at 500 °C for 20 min
Cheng et al. (2023) [105]	EP/CF	Thermally activated Cr_2_O_3_ at 520 °C for 35 min in air	Nearly complete resin removal; no evident carbon deposition; clean filamentous surface with increased C–OH and COOH groups	Strength: 94.62%; Elastic modulus: 97.37%
Cheng et al. (2023) [108]	EP/CF	Cr_2_O_3_-assisted thermal treatment at 360–520 °C for 5–30 min under O_2_	Resin residue decreased with temperature and time; fibers became cleaner at higher O_2_ concentration, accompanied by increased surface oxidation and COOH formation	Strength: 83.3–130.3% apparent retention, depending on temperature, time, and O_2_ concentration
Feng et al. (2023) [91]	Hemiacetal ester dynamic covalent polymer/CF	Immersion in 0.1 M HCl/acetone solution (9:1) at 25 °C; solution renewed every 8 h; acetone washing and drying at 75 °C for 24 h	Clean and smooth fiber surface without obvious matrix residue, damage, or dimensional change; Raman and XPS responses were nearly unchanged	Strength: 94.9%; Elastic modulus: 97.7%; Elongation: 94.1%
Ren et al. (2023) [116]	EP/CF	Ar pyrolysis at 550 °C for 30 min, followed by oxidation at 499 °C for 10 min under 30% O_2_	Clean and smooth surface without visible resin or char; longitudinal grooves retained; roughness decreased from 133 nm for the pyrolytic solid to 85 nm for rCF, compared with 102 nm for vCF	Strength: 96.15%; Elastic modulus: 102.15%
Wei & Hadigheh (2023) [81]	EP/CF	0.1 N acetic acid heated to 100 °C without isothermal dwelling; pyrolysis to 425 °C and oxidation to 550 °C at 15 °C/min	Clean and smooth surface without visible matrix or char; the 80 °C/60 min pretreatment caused local fiber damage	Strength: 90.53%, 80.32% when thermal-only; Elastic modulus: 95.0%.
Cheng et al. (2022) [109]	EP/CF	Cr_2_O_3_-coated honeycomb at 500 °C for 25 min under 80% O_2_/N_2_; 180 mL/min	EP decomposition: 95.5%; very little residue and no carbonaceous solid; slight surface oxidation with increased C–OH and COOH groups	Monofilament tensile properties maintained more than 97%
Guo et al., (2022) [132]	EP/CF	Ar pyrolysis at 600 °C for 30 min, followed by air oxidation at 450 °C for 15–20 min.	Carbon residue remained after pyrolysis; oxidation at 450 °C for 15–20 min produced relatively smooth fibers without obvious damage, whereas 10 min left residual carbon and excessive oxidation promoted surface degradation.	Strength: exceeds 90.19%; Elastic modulus: exceeds 84.44%
Guo et al. (2019) [104]	EP/CF	Vacuum pyrolysis at 900 °C for 1 h; heating rate of 10 °C/min	Needle-felt structure largely retained; discontinuous pyrolytic char adhered to the fibers and locally bonded adjacent filaments	Strength: 97.44%; Elastic modulus: 98.70%
Tapper et al. (2019) [90]	PA6/CF	Benzyl alcohol dissolution and acetone precipitation over two recycling loops	Residual PA6 coating, fiber shortening, agglomeration, and increasing misalignment; near-original 3 mm fibers decreased from 63.9% to 38.6% and 15.9%	Strength 61.2%59.6%; Elastic modulus: 75.4/60.3%; Rupture strain 94.8/96.6% (at the 1st/2nd loop)
Sun et al. (2015) [76]	EP/CF	NaCl electrolysis for 21 d; 3–20% NaCl; 4–25 mA	Clean and crack-free under mild conditions; higher NaCl concentration intensified oxidation and chlorination	Strength: 32–80%
